# OsCPN10a, cooperating with OsCPN20 and OsHSP60‐3B negatively regulate ABA signaling and enhance seed storability in rice

**DOI:** 10.1111/jipb.70230

**Published:** 2026-03-16

**Authors:** Sufeng Liao, Yidong Wei, Yongsheng Zhu, Kunyang Li, Ting Chen, Shuai Zhao, Fangxi Wu, Jinlan Wang, Pengbo Yu, Hongguang Xie, Liping Chen, Qiuhua Cai, Huaan Xie, Jianfu Zhang

**Affiliations:** ^1^ Rice Research Institute Fujian Academy of Agricultural Sciences Fuzhou 350019 China; ^2^ Cross‐straits Agricultural Technology Cooperation Center, College of Agriculture Fujian Agriculture and Forestry University Fuzhou 350002 China; ^3^ State Key Laboratory of Ecological Pest Control for Fujian and Taiwan Crops, Key Laboratory of Germplasm Innovation and Molecular Breeding of Hybrid Rice for South China Ministry of Agriculture and Affairs Fuzhou 350003 China; ^4^ Incubator of National Key Laboratory of Germplasm Innovation and Molecular Breeding between Fujian and Ministry of Sciences and Technology, Fujian Engineering Laboratory of Crop Molecular Breeding, Fujian Key Laboratory of Rice Molecular Breeding National Rice Improvement Center of China Fuzhou 350003 China; ^5^ Key Laboratory for Basic and Applied Research of Bayu Prescriptions and Herbs, College of Chinese Materia Medica Chongqing University of Chinese Medicine Chongqing 361000 China

**Keywords:** ABA signaling, molecular chaperone, *Oryza sativa*, *OsCPN10a*, ROS homeostasis, seed storability, starch integrity

## Abstract

Seed storability is crucial for maintaining vigor at the sowing stage and ensuring agricultural sustainability. Although molecular chaperones are well characterized in protein homeostasis, their roles in regulating ABA receptor stability and starch integrity during seed aging remain unclear. Here, we demonstrate that *OsCPN10a* enhances storability by maintaining ABA homeostasis, suppressing ROS accumulation, and preserving starch granule structure. Genetic and biochemical analyses reveal that OsCPN10a functions upstream of the OsCPN20–OsHSP60‐3B cascade, directly interacts with both partners to promote complex assembly, and negatively regulates ABA signaling. The resulting trimeric complex interacts with OsPYL10, facilitating receptor dissociation and high‐affinity association with the PP2C phosphatase OsABIL1 to attenuate signaling. This study reveals a non‐canonical function of molecular chaperones in directly modulating hormone receptor signaling, establishing a mechanistic link between protein homeostasis and ABA sensitivity in seeds.

## Graphical abstract

Abscisic acid signaling homeostasis is essential for seed storability. The molecular chaperone OsCPN10a enhances rice seed storability by forming a trimeric chaperone complex with OsCPN20‐OsHSP60‐3B that attenuates abscisic acid signaling via direct interaction with OsPYL10‐OsABIL1, thereby maintaining starch integrity and offering a promising target for crop improvement.

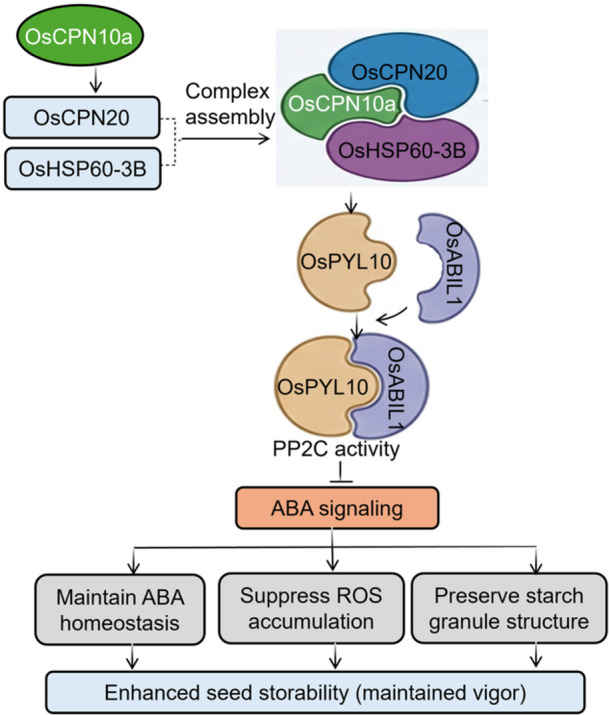

## INTRODUCTION

Seed storability of rice (*Oryza sativa* L.) is a crucial determinant of seed quality, determining the capacity of the embryo to maintain viability to germinate and genetic integrity into healthy seedlings after long‐term storage (Yan et al., 2024; Kannababu et al., 2025). In the long‐term preservation system of germplasm banks, high storability can extend the half‐life of the germination rate attenuation to over 30 years, markedly reducing the frequency of regeneration cycles, cutting conservation costs by 20%–40%, and guaranteeing an emergency seed reservoir under extreme climatic shocks (Ding et al., 2023). Beyond conservation, enhanced seed vigor directly translates to agricultural productivity, with germination improvements potentially increasing yields by 20%–50% in major cereals, including rice, wheat, and maize (Tester and Langridge, 2010). Nevertheless, seeds inevitably undergo progressive aging during storage, manifested as rapid viability decline, non‐uniform seedling emergence, and compromised stress tolerance (Naghisharifi et al., 2024). Consequently, identifying genetic determinants governing seed storability and deciphering their regulatory networks are imperative for breeding rice cultivars with extended storage life and stable field performance.

Seed storability is a complex, multifactorial trait governed by diverse physiological and molecular mechanisms (Zhou et al., 2019; Han et al., 2022). Recent advances in genomics, transcriptomics, and proteomics have facilitated the fine‐mapping and cloning of quantitative trait loci (QTLs) associated with rice seed storability and vigor. Major QTLs specifically controlling storability include *RC7*, *RC9‐1*, *RC9‐2*, *RC9‐3*, *qLG‐2*, *qLG‐4*, *qLG‐9*, *qLS‐9*, *qLS‐11*, and *qLS‐12* (Sasaki et al., 2005; Zeng et al., 2006; Fujino et al., 2008). Additionally, several genes with pleiotropic effects on both traits, including *qSD1–2/OsGA20ox2, OsGA20ox1*, *OsFbx352*, *OsPIPI*, *OsLOL1*, *OsPP2C68*, and *OsPYL/RCAR10*, have been characterized (Abe et al., 2012; Kim et al., 2012; Song et al., 2012; Liu et al., 2013; Wu et al., 2014; Tian et al., 2015; Li et al., 2016). Notably, among these, Abscisic acid (ABA) signaling integrates seed maturation, dormancy, and storability through core components such as ABIs, PYL/RCAR receptors, and PP2C phosphatases (Bhatnagar et al., 2017; Ali et al., 2022).

Ultimately, these genetic networks safeguard storage reserves, particularly starch, which serve as the primary energy source in the endosperm (Zhang et al., 2021; Hu et al., 2022; Wang et al., 2022). However, the genetic determinants ensuring starch stability during prolonged storage remain poorly understood. Moreover, while these QTLs provide a valuable genetic foundation, most lack functional validation due to large genomic intervals containing multiple candidates and complex gene‐environment interactions (Manichaikul et al., 2009; Han et al., 2022). Consequently, precise characterization of causal genes is essential to translate this genetic knowledge into practical breeding applications.

During dry storage, seeds undergo drastic dehydration, often reaching below 5% moisture content. This leads to the accumulation of denatured proteins and aberrant protein aggregates, particularly within plastids where metabolic preparations for germination initiate (Catusse et al., 2008; Rajjou et al., 2012). Emerging evidence positions proteostasis capacity as a central pillar of seed longevity, wherein aging tolerance depends on the coordinated action of heat shock proteins (HSPs) and associated chaperone systems (Wu et al., 2022b). Plant HSPs comprise five major families classified by molecular mass: HSP100/ClpB, HSP90, HSP70/DnaK, HSP60/chaperonins, and ATP‐independent small HSPs (de Jongh et al., 1998; Sarkar et al., 2013; Shi et al., 2016; Sadat et al., 2022). These families contain multiple isoforms targeted to distinct subcellular compartments (cytosolic, chloroplastic, mitochondrial, and endoplasmic‐reticulum), enabling compartment‐specific protection during seed development, desiccation, and aging. Among these, small HSPs (10–42 kDa) constitute the most diverse family, characterized by a conserved α‐crystallin domain (ACD) and seed‐specific expression patterns (de Jongh et al., 1998). In rice, 23 small HSPs have been identified, the majority exhibiting seed‐specific expression suggestive of specialized roles in seed physiology (Chen et al., 2014; Guo et al., 2020; He et al., 2021; Wu et al., 2022b).

Within the chaperone network, chaperonins are critical for maintaining organellar proteostasis. For instance, the type I chaperonin CPN60 and its co‐chaperonin CPN10 form a unique ATP‐dependent folding compartment essential for the maturation of approximately 15%–20% of plastidic proteins. These substrates include the large subunit of ribulose‐1,5‐bisphosphate carboxylase/oxygenase and key enzymes involved in fatty acid and amino acid metabolism (Cloney et al., 1992; Ke et al., 2017). Unlike the HSP70/HSP90 systems, which operate through cycles of substrate binding and release in an open environment, the CPN60 complex forms a double‐ring barrel structure that encapsulates unfolded substrates within a central cavity, with CPN10 serving as the detachable lid essential for completing the folding chamber (Hartl and Hayer‐Hartl, 2009). Loss of CPN10 function leaves CPN60 barrels open, resulting in the accumulation of aggregation‐prone intermediates that compromise cellular integrity (Kim et al., 2013; Balchin et al., 2020). Notably, *OsHSP60‐3B* controls amyloplast development and starch granule biogenesis in rice pollen, indicating conserved roles for chaperonins in storage organelle function (Lin et al., 2023). Nevertheless, whether these plastidic chaperonin systems protect amyloplasts and maintain starch stability during the prolonged quiescence of seed aging remains unknown.

Interestingly, chaperonins also mediate hormone signaling. In *A. thaliana*, the chloroplast chaperonin AtCPN20 forms complexes with ABA receptor CHLH/ABAR to negatively regulate ABA signaling (Zhang et al., 2014). Although these findings illuminate chaperonin‐hormone crosstalk in vegetative tissues, the role of proteostatic machinery in ABA‐mediated seed aging remains obscure. While upstream ABA signaling components (OsPYL/RCAR receptors, OsPP2Cs) regulate seed dormancy, whether chaperonin functions as a downstream effector to maintain organellar proteostasis during seed aging remains unknown.

Here, we identify *OsCPN10a*, a 10 kDa co‐chaperonin, as a positive regulator of seed storability whose expression is transcriptionally responsive to ABA. Through functional characterization using clustered regularly interspaced short palindromic repeats (CRISPR)/CRISPR‐associated protein 9 (Cas9)‐mediated knockout and overexpression lines, we demonstrate that OsCPN10a is indispensable for maintaining the antioxidant enzyme activities and starch structure integrity during seed aging. We further reveal that OsCPN10a forms a functional complex with OsCPN20 and OsHSP60‐3B, and this chaperonin machinery unexpectedly functions beyond protein folding to stabilize the interaction between the ABA receptor OsPYL10 and the phosphatase OsABIL1. This fine‐tuning of ABA signaling sensitivity enables precise control of seed vigor. Thus, our findings reveal a novel molecular framework to regulate seed vigor. More importantly, we provide a potential strategy for simultaneously improving both seed aging tolerance and vigor by designing *OsCPN10a* directionally.

## RESULTS

### OsCPN10a regulates seed storage tolerance in rice

**Figure 1:**
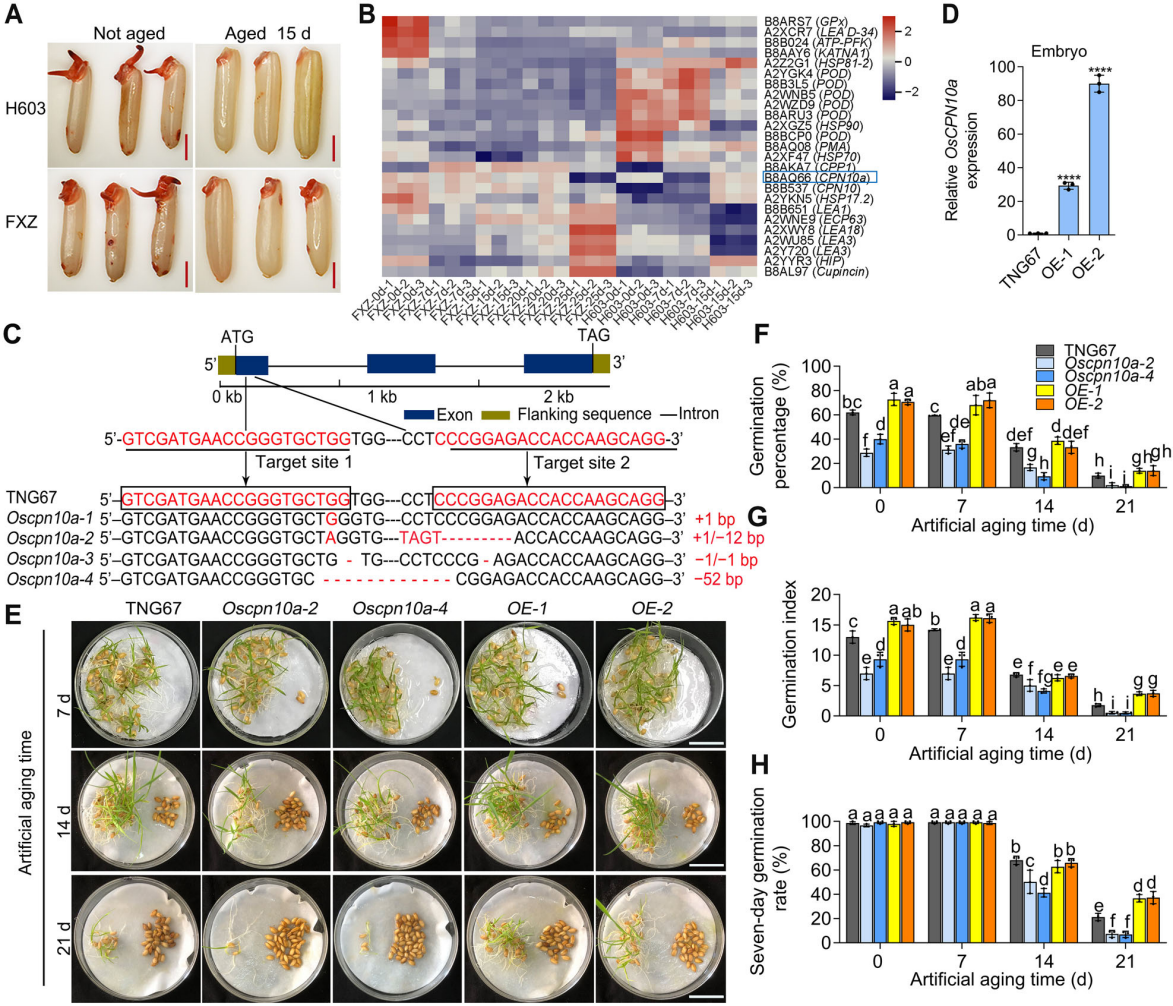
***OsCPN10a*** **positively regulates resistance to seed aging** **(A)** 2,3,5‐Triphenyltetrazolium chloride (TTC) staining of the seed embryos of rice cultivars H603 and Fuxiangzhan (FXZ) before (Not aged) and after (Aged 15 d) artificial aging treatment. Scale bar, 0.5 cm. **(B)** Heatmap showing the differential accumulation of heat shock proteins (HSPs) and their associated proteins in H603 and FXZ seed embryos under artificial aging, as determined by iTRAQ‐based comparative proteomic analysis. Protein abundance fold changes were visualized and analyzed using TBtools software (version 2.012). Color gradients represent log₂‐transformed fold‐change values, with red indicating upregulation, blue indicating downregulation, and white representing no significant change. **(C)** Base mutation site of *OsCPN10a*. The knockout target sites of *OsCPN10a* are marked in red base sequence. Sequences in boxes are the target sequences in wild‐type TNG67. Red dashes represent deleted bases, and red uppercase letters indicate inserted nucleotides. **(D)** Relative *OsCPN10a* expression in two *OsCPN10a* overexpression lines (*OE‐1* and *OE‐2*) in the TNG67 background versus the wild‐type. *OsACTIN150* was used as the internal control. Data are given as means ± *SD* from three biologically independent replicates (*n* = 3; *P < 0.05, **P < 0.01; Student's *t*‐test). **(E)** Photographs of germinated seeds from wild‐type TNG67, *oscpn10a* mutant (*Oscpn10a‐2* and *Oscpn10a‐4*), and *OsCPN10a* overexpression (*OE‐1* and *OE‐2*) lines taken at 7 d post‐imbibition after artificial aging for 7, 14, or 21 d. Scale bar, 2 cm. **(F**–**H)** Assessment of aging resistance in wild‐type TNG67, *Oscpn10a* mutants, and *OsCPN10a* overexpression lines. Data are given as means ± *SD* of three biological replicates (50 seeds per biological replicate). Different lowercase letters above the bars indicate significant differences (*P* < 0.05) based on two‐way analysis of variance (ANOVA).

Fuxiangzhan (FXZ) is an elite *Indica* cultivar with high tolerance to seed storage, whereas its parental line H603 is an *Aus* cultivar with low seed storage tolerance (Liao et al., 2025). To assess the impact of artificial aging on embryonic viability, 2,3,5‐triphenyltetrazolium chloride (TTC) staining was performed on the embryos of FXZ and H603 seeds after artificial aging treatment. After 15 d of artificial aging, H603 embryos showed markedly paler TTC staining in the coleorhiza, radicle, coleoptile, and scutellum relative to unaged controls, while that of the indica variety FXZ only slightly decreased (Figure 1A), indicating severe loss of embryonic viability in H603 and confirming the superior storability of FXZ. Given these phenotypic differences in storability and embryonic viability, an iTRAQ‐based comparative proteomic analysis was previously conducted to examine the changes in protein accumulation level in embryos of aged FXZ and H603. After the 15‐d artificial aging period, H603 embryos exhibited significantly reduced accumulation of aging marker proteins, including late embryogenesis abundant proteins and chloroplast metalloprotease 1, compared with untreated controls. In contrast, the abundance of antioxidant‐related proteins, specifically glutathione peroxidase and peroxidase (POD), was significantly upregulated (Figure 1B). These changes are consistent with the weak storability of H603. Notably, the abundance of several HSP family proteins was highly correlated with the differential accumulation of the aforementioned proteins, with B8AQ66 (hereafter designated OsCPN10a) being the most prominent (Figure 1B). OsCPN10a protein was significantly downregulated in H603 after 7 d of artificial aging, but only exhibited significant downregulation in FXZ after 25 d of aging, suggesting a potential association with rice embryonic viability.

To determine the basis of the differential accumulation of OsCPN10a protein between FXZ and H603, gene cloning was conducted, which confirmed that the two cultivars share an identical *OsCPN10a* sequence. This implies that the observed protein level differences may arise from transcriptional regulation. The reverse transcription quantitative real‐time PCR (RT‐qPCR) analysis of *OsCPN10a* revealed upregulation in both FXZ and H603 after 7 d of artificial aging, although after 15 d, transcript levels had declined in H603, while remaining high in FXZ (Figure S1A). This transcriptional trend was consistent with the protein level changes, further supporting a role for *OsCPN10a* in regulating embryonic viability. To validate the universality of *OsCPN10a* expression patterns, representative cultivars with distinct storability were analyzed. Following 15 d of artificial aging, highly storable cultivars, including *Aus* cultivars Yunhui 290, 93‐11, Yuejingsimiao, Qingjingzhan, FXZ, Huazhan, Yujingyouzhan, 676, and Yuzhenxiang, maintained high germination rates (> 80.00%) alongside generally elevated *OsCPN10a* expression levels (Figure S1B, C). In contrast, low‐storability cultivars such as Nipponbare, Tainong 67 (TNG67), Minghui 86, and H603 exhibited relatively low *OsCPN10a* expression in embryos (Figure S1B, C). Extending this analysis to transcriptional levels before and after aging across all tested cultivars, a significant positive correlation with seed germination rates was observed (*r* = 0.59, *P* < 0.01) (Figure S1D). Collectively, these results indicate that *OsCPN10a* may be involved in the regulation of embryonic viability.

To further verify the role of *OsCPN10a* in regulating seed storage tolerance, we generated *OsCPN10a* knockout plants (*Oscpn10a‐1*, *Oscpn10a‐2*, *Oscpn10a‐3*, and *Oscpn10a‐4*) using CRISPR/Cas9 genome editing technology in the TNG67 background. *Oscpn10a‐1* had a 1‐bp insertion, while *Oscpn10a‐2, Oscpn10a‐3*, and *Oscpn10a‐4* harbored 11‐bp, 2‐bp, and 52‐bp deletion, respectively (Figures 1C, S2). Meanwhile, *OsCPN10a* overexpressed lines were constructed in TNG67. Significant accumulation of *OsCPN10a* transcripts in the embryo of *OsCPN10a* overexpressed lines (*OE‐1* and *OE‐2*) was confirmed by RT‐qPCR (Figure 1D). Despite *Oscpn10a* seeds showing 100% germination under normal conditions, the 4‐d germination percentage (GP) of newly harvested *Oscpn10a‐2* and *Oscpn10a‐4* seeds was only 40.00% and 18.67%, respectively, compared with 62.00% for TNG67, whereas *OE‐1* and *OE‐2* reached 72.67% and 70.67%, respectively (Figure 1E, F, H). Dormancy‐breaking treatment partially restored the germination performance of *Oscpn10a* mutant seeds, yet their GP was still significantly lower than that of TNG67 and *OsCPN10a* overexpressing seeds (Figure S3), indicating that knockout of *OsCPN10a* at least partially prolongs the germination process by regulating the seed dormancy pathway. After 21 d of artificial aging treatment, seeds from *Oscpn10a* mutants exhibited a sharp decrease in GP, germination index (GI), and 7‐d germination rate (GR) than TNG67 (Figure 1E, F, H). In contrast, the *OsCPN10a* overexpression lines maintained higher GR compared to TNG67 (Figure 1H). After aging naturally for 6 months, the GP, GI, and GR of *Oscpn10a* mutant seeds were also significantly lower than those of TNG67 (Figure S4A–D). However, in the *OsCPN10a* overexpression lines, these measures exceeded those of the TNG67 (Figure S4A–D). Following heat stress treatment at 38°C for 7 d, *Oscpn10a* mutants showed significantly reduced germination compared to wild‐type and overexpression lines (Figure S4E–H). Notably, more than 70% of TNG67, *OE‐1*, and *OE‐2* seeds had germinated, whereas only 63.09% and 58% of *Oscpn10a‐2* and *Oscpn10a‐4* seeds had germinated, respectively (Figure S4G). Taken together, these results suggest that *OsCPN10a* regulates both seed storage tolerance and heat tolerance in rice.

### *OsCPN10a* regulates seed storability by reducing ROS levels and enhancing antioxidative activity

**Figure 2:**
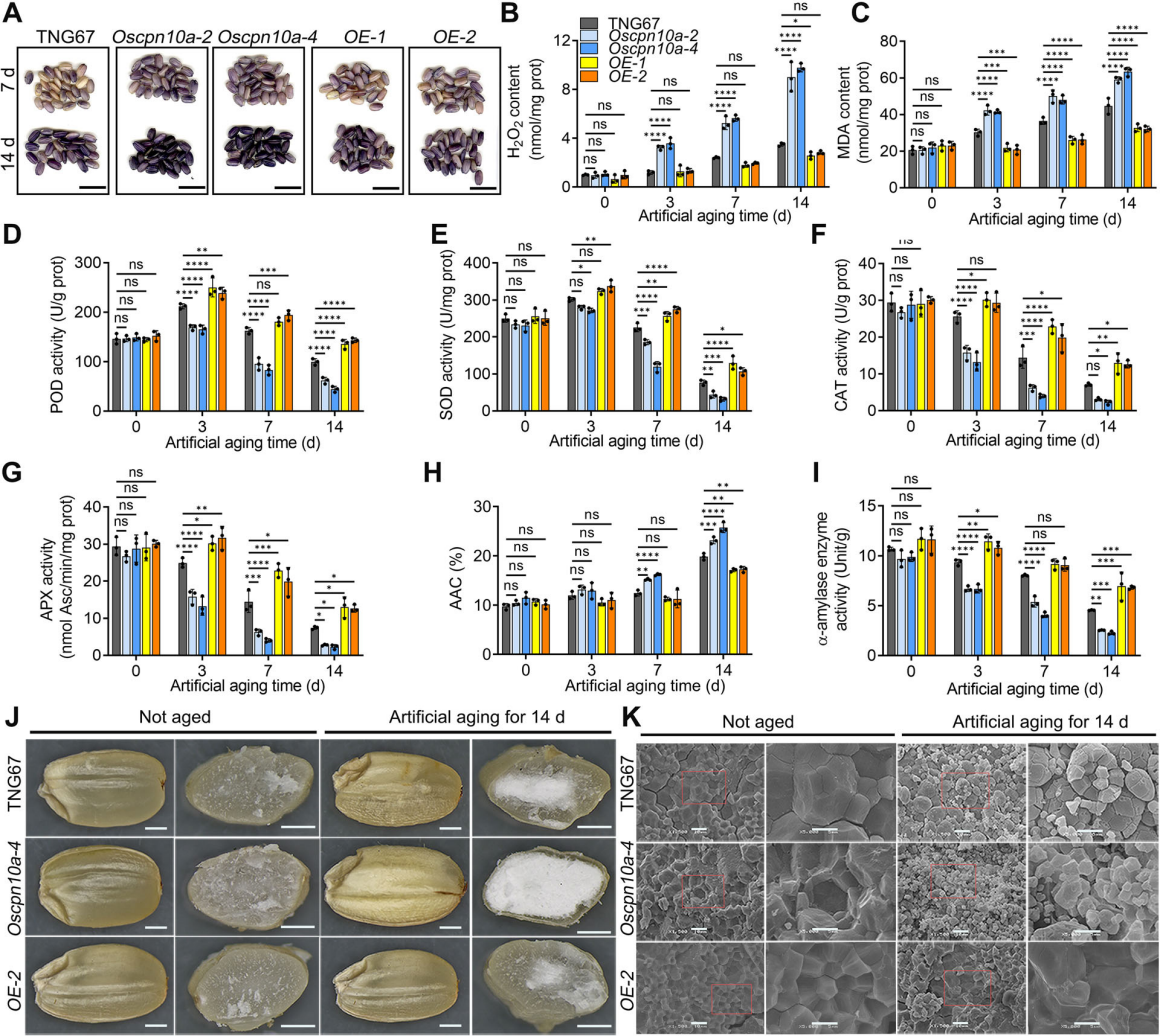
***OsCPN10a*** **suppresses aging‐induced starch structure disorder and enhances antioxidant activity** **(A)** NBT staining for H_2_O_2_ production in seeds from wild‐type TGN67, *Oscpn10a* mutant, and *OsCPN10a* overexpression lines after 7 and 14 d of artificial aging. Scale bar, 1 cm. **(B**, **C)** H_2_O_2_ **(B)** and malondialdehyde (MDA; **C**) contents in embryos of wild‐type TGN67, *Oscpn10a* mutant, and *OsCPN10a* overexpression lines. **(D**–**G)** Peroxidase (POD; **D**), superoxide dismutase (SOD; **E**), catalase (CAT; **F**), and ascorbate peroxidase (APX; **G**) activities in embryos of wild‐type TGN67, *Oscpn10a* mutant, and *OsCPN10a* overexpression lines. **(H)** Apparent amylose content (ACC) in embryos of wild‐type TGN67, *Oscpn10a* mutant, and *OsCPN10a* overexpression lines. **(I)** α‐Amylase activity in embryos of wild‐type TGN67, *Oscpn10a* mutants, and *OsCPN10a* overexpression lines. In **(B**–**I)**, data are given as means ± *SD* from three biologically independent replicates (*n* = 3). Student's *t‐*test was used to generate the *P*‐values: *P < 0.05, **P < 0.01, ***P < 0.001, ****P < 0.0001. **(J)** Grain morphology and cross‐sections of wild‐type TNG67, *Oscpn10a* mutant, and *OsCPN10a* overexpression lines before and after 14 d of artificial aging. Scale bar, 100 μm. **(K)** Scanning electron micrographs of cross‐sections of wild‐type TNG67, *Oscpn10a*‐4, and *OE‐2* grains after 14 d of artificial aging. Boxed regions in the first and third columns are enlarged in the adjacent images in the second and fourth columns. Scale bars, 10 μm in the first and third columns and 5 μm in the second and fourth columns.

Seed aging is accompanied by ROS accumulation, which adversely affects cellular proteins and enzymes (Jimenez et al., 2025). Thus, we first investigated whether *OsCPN10a* overexpression protects seeds against ROS‐mediated damage. Nitro blue tetrazolium (NBT) staining was used for this observation as it stains areas of H_2_O_2_ production. The results clearly demonstrated that the *Oscpn10a* endosperms accumulated more H_2_O_2_ after 7 and 14 d of artificial aging compared with TNG67, whereas the *OsCPN10a* overexpression lines accumulated less H_2_O_2_ content (Figure 2A). Consistently, *Oscpn10a* mutants exhibited a 2.6‐ to 2.8‐fold increase in H_2_O_2_ and a 1.32‐ to 1.42‐fold rise in malondialdehyde (MDA) content relative to TNG67 after 14 d of artificial aging, whereas the *OsCPN10a* overexpression lines maintained both markers at control levels (Figure 2B, C). This oxidative burst coincided with a systematic suppression of antioxidant capacity: superoxide dismutase (SOD), POD, catalase (CAT), and APX activities in the *Oscpn10a* mutants were reduced to 30.54%–62.17% of those in TNG67 at the same time points, whereas the *OsCPN10a* overexpression embryos displayed the opposite trend (Figure 2D, E, G). These data indicated that *OsCPN10a* enhanced antioxidative activity and reduced ROS levels during seed aging.

During seed aging, ROS accumulation oxidatively modifies amylases and lowers their activity, thereby decelerating the enzymatic hydrolysis of starch granules (Chen, 2023). Thus, we analyzed the apparent amylose contents (AAC) of endosperms. After 14 d of artificial aging, the AAC content of *Oscpn10a‐2* and *Oscpn10a‐4* endosperms was significantly higher than that of TNG67, whereas the *OsCPN10a* overexpression lines exhibited markedly reduced AAC (Figure 2H). Conversely, after 3, 7, or 14 d of artificial aging, endosperms from *OsCPN10a* overexpression lines displayed a marked increase in α‐amylase activity upon imbibition, while the *Oscpn10a* mutants retained < 45% of α‐amylase activity at the same time points compared with TNG67 (Figure 2I). In addition, stereomicroscope analysis of *Oscpn10a‐4* endosperm revealed significantly increased chalky areas, while the *OsCPN10a* overexpression line (*OE‐2*) exhibited a significant reduction in these traits, compared with the wild‐type TNG67 (Figure 2J). Additionally, observations of cross‐sections by scanning electron microscopy showed that, after artificial aging for 14 d, *Oscpn10a‐4* endosperm exhibited a marked increase in the number of small starch granules, a decrease in the number of large starch granules, accompanied by fewer equatorial furrows and micropores on the granule surface compared to TNG67 (Figure 2K). In contrast, *OE‐2* showed a significant reduction in small starch granules (Figure 2K). Together, these data demonstrate that *OsCPN10a* is essential for maintaining seed starch‐granule integrity during seed aging.

### *OsCPN10a* is highly expressed in young tissues and localizes in the nucleus and endoplasmic reticulum to function as a molecular chaperone

**Figure 3:**
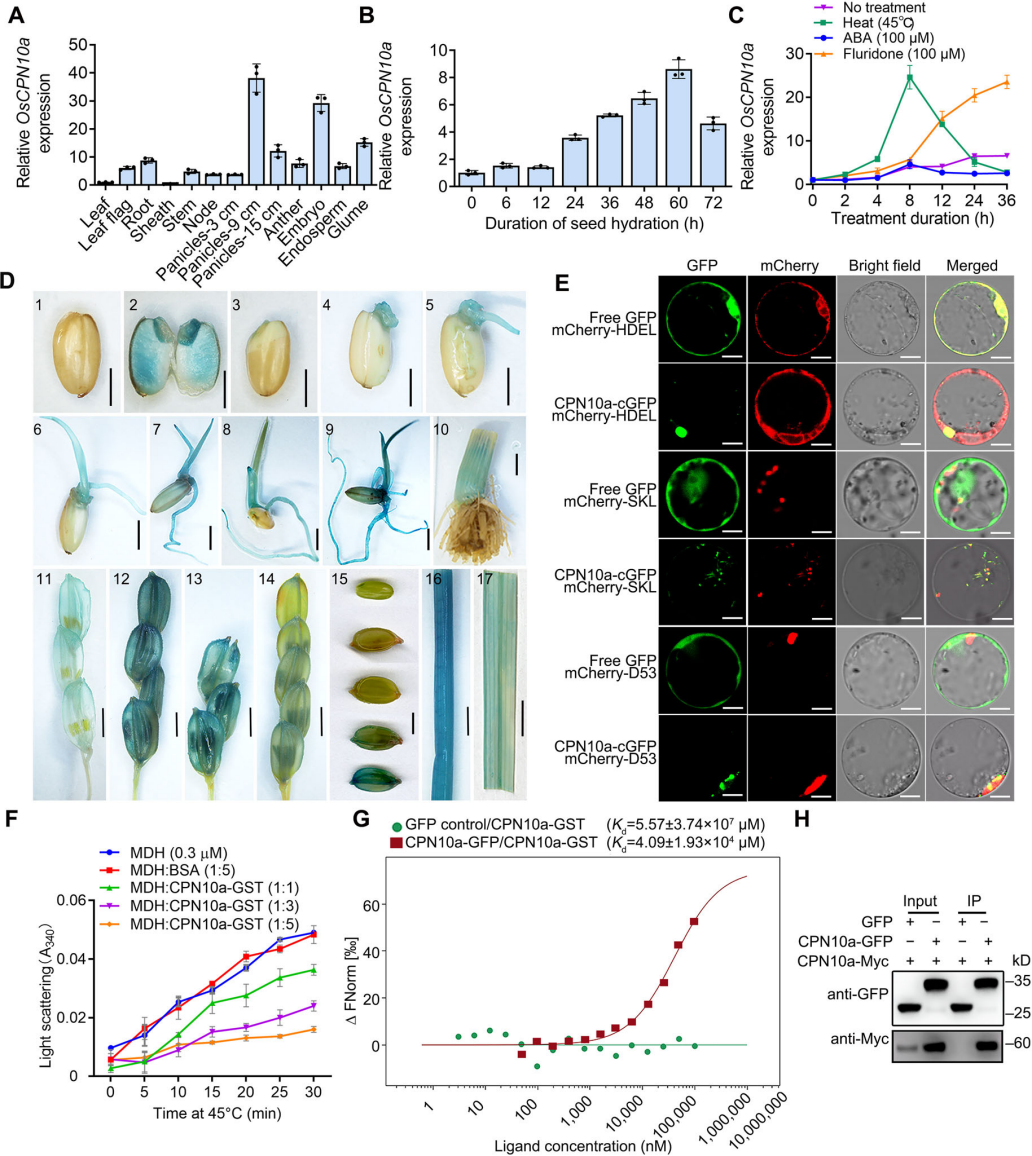
**Expression pattern and chaperone activity of** ***OsCPN10a*** **(A)** Analysis of *OsCPN10a* expression in various organs by reverse transcription quantitative real‐time PCR (RT‐qPCR) in TNG67 plants. **(B)** Relative *OsCPN10a* expression in germinated TNG67 embryos at 0, 6, 12, 24, 30, 36, 48, 54, 60, and 72 h after the duration of seed hydration. **(C)** Relative expression of *OsCPN10a* was determined by RT‐qPCR immediately after 0, 2, 4, 8, 12, 24, and 36 h of heat (45°C), abscisic acid (100 μM ABA), or fluridone (100 μM) treatment. In **(A**–**C)**, data are given as means ± *SD* from three biologically independent replicates (*n* = 3). *OsACTIN150* as the internal control. **(D)** GUS activity was detected in the panicles and spikelets of *proOsCPN10a*::*GUS* transgenic plants at the following developmental stages: Germinating seedlings at 0 d (1), 1 d (2, 3), 2 d (4), 3 d (5), 4 d (6), 5 d (7), 7 d (8), and 10 d (9) after imbibition; the root head (10); flowers and rice grains in the filling stage, milk ripening stage, and wax ripening stage (11–15); stem (16); and leaf (17). Scale bar, 1 cm. **(E)** Subcellular localization of OsCnp10a‐GFP in rice protoplasts. OsCPN10a‐GFP, tagged at the C terminus with GFP, was co‐expressed with mCherry‐HDEL (endoplasmic reticulum marker), mCherry‐SKL (peroxisome marker), or D53‐mCherry (nuclear marker) in rice protoplasts, independently. Scale bar, 10 μm. **(F)** Chaperone activity assay of OsCPN10a protein. Chaperone activity was measured as the ability to prevent MDH denaturation under thermal‐denaturing conditions at 45°C. BSA was used as a negative control. Data are presented as means ± *SD* from three biologically independent replicates (*n* = 3). **(G)** Microscale thermophoresis test (MST) confirmed that OsCPN10a forms homo‐oligomers that act as a molecular chaperone *in vitro*. The normalized fluorescence change (ΔFNorm [‰]) was plotted against ligand concentration. Data represent means ± *SD* from three independent experiments (biological replicates), with 16 serial dilutions (technical replicates) per experiment. Representative binding curves are shown. **(H)** Co‐IP assay verified the interaction between OsCPN10a‐GFP and OsCPN10a‐Myc. IP, immunoprecipitation; Anti‐GFP and anti‐Myc indicate two labeled antibodies. The experiment was repeated three times with consistent results.

To characterize the function of *OsCPN10a*, phylogenetic analysis was performed. The results revealed that *OsCPN10a* belongs to the GroES/Cpn10 family (Figure S5), contains a conserved 91‐residue *α*‐crystal domain at the C‐terminal essential for sHSPs oligomerization (Figure S6). RT‐qPCR revealed *OsCPN10a* to be widely expressed in 10‐d leaf flags, anthers, 10‐d roots, and glumes, with the highest transcript level detected in 9‐cm panicles, followed by embryos (Figure 3A). During the early stage of seed germination, *OsCPN10a* transcripts began to accumulate at 24 h after imbibition, peaking at 60 h in embryos (Figure 3B). During early germination, *OsCPN10a* expression reached a peak of 9.45‐fold and 24.2‐fold in embryos treated with abscisic acid (ABA, 24 h) or heat (8 h), respectively. However, a continuous induction was observed under fluridone (an ABA biosynthesis inhibitor) treatment, with its expression level significantly higher compared to the control group (Figure 3C). We further generated transgenic rice plants expressing the GUS (*β*‐glucuronidase) reporter gene driven by the *OsCPN10a* promoter and examined the *OsCPN10a* expression profile during spikelet development and seed germination. Histochemical staining revealed that the strongest signal was observed in the spikelet hulls, milk‐stage grains, and stems, followed by the leaf. Furthermore, *OsCPN10a* was strongly expressed in the spikelet meristems with a gradual decrease as development progressed (Figure 3D). During early seed germination, GUS activity was observed predominantly in the emerging radicle, endosperm, and embryo of germinated seeds at 24 h after imbibition (Figure 3D, 2–5) and became more obvious in 7‐, 8‐, and 9‐d‐old seedlings (Figure 3D, 6–9). These findings were consistent with the qPCR results. Thus, *OsCPN10a* is preferentially expressed in the young tissues during seed development and germination, supporting its role in regulating embryo viability and germination capacity in rice as presented above.

We next analyzed the subcellular localization of OsCPN10a and found that OsCPN10a colocalized in the nucleus with the nuclear marker D53‐mCherry in rice protoplasts (Zhou et al., 2013; Figure 3E). In addition, OsCPN10a partially colocalized with the endoplasmic reticulum marker mCherry‐HDEL and peroxisome marker mCherry‐SKL (Figure 3E). To determine the biochemical characteristics of OsCPN10a as a molecular chaperone, an OsCPN10a‐GST fusion protein was expressed and purified from *Rosetta* (*DE3*) *Escherichia coli strains*, and its effect on the aggregation rate of malate dehydrogenase (MDH) at 45°C was assessed. Recombinant OsCPN10a significantly inhibited MDH aggregation under high‐temperature conditions, with maximum inhibitory efficiency achieved at an MDH:OsCPN10a molar ratio of 1:5 (Figure 3F), indicating that OsCPN10a may function as a molecular chaperonin *in vivo*. To clarify how OsCPN10a exerts its chaperone function, OsCPN10a‐GFP expressed in rice protoplasts and OsCPN10a‐GST expressed in *DE3* were used for microscale thermophoresis (MST) assays, which demonstrated that OsCPN10a is capable of self‐interaction (Figure 3G). This self‐interaction was further confirmed by co‐immunoprecipitation (Co‐IP) assay showing that OsCPN10a‐Myc protein specifically interacts with OsCPN10a‐GFP protein (Figure 3H). These results showed that OsCPN10a functions as a homodimer to exert its molecular chaperone role.

### OsCPN10a accelerates seed germination by negatively regulating ABA signaling

**Figure 4:**
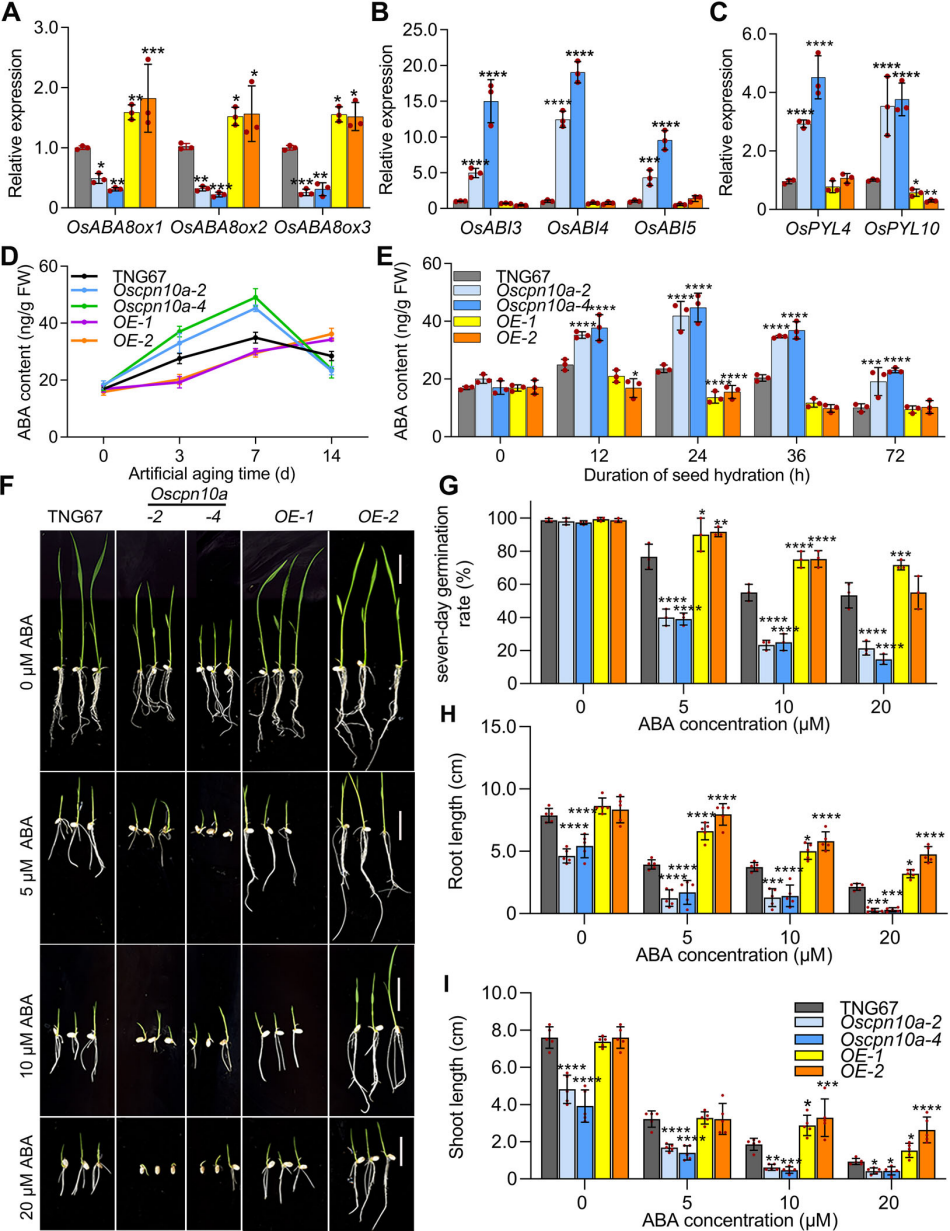
***OsCPN10a*** **regulates seed germination by modulating ABA metabolism and signal transduction** **(A**–**C)** Relative expression of ABA degradation genes (*OsABA8ox1/2/3*) and ABA signaling genes (*OsABI3/4/5* and *OsPYL4/10*) in embryos of wild‐type TNG67, *Oscpn10a* mutant, and *OsCPN10a* overexpression lines. Relative expression is represented by the fold‐change relative to expression in TNG67. *Actin150* was used as the internal control. **(D)** ABA content in embryos of TNG67, *Oscpn10a* mutant, and *OsCPN10a* overexpression lines during 0–14 d of artificial aging. **(E)** ABA content in embryos of wild‐type TNG67, *Oscpn10a* mutant, and *OsCPN10a* overexpression lines during 0–72 h after imbibition. **(F)** Representative images of germinated seeds from wild‐type TNG67, *Oscpn10a* mutant, and *OsCPN10a* overexpression lines at 6 d after imbibition under 0, 5, 10, or 20 μM ABA treatment. Scale bars, 15 mm. **(G**–**I)** Germination rate, root length, and shoot length of wild‐type TNG67, *Oscpn10a* mutant, and *OsCPN10a* overexpression lines after 6 d of imbibition in response to treatment with the indicated concentrations of ABA. In **(A**–**E**, **G)**, data are given as means ± *SD* from three biological replicates (*n* = 3). In **(H**, **I)**, data are presented as means ± *SD* from five biological replicates (*n* = 5). *P < 0.05, **P < 0.01, ***P < 0.001, ****P < 0.0001 (two‐tailed ANOVA).

To identify downstream genes regulated by *OsCPN10a*, we examined the transcript levels of key genes regulating seed germination in embryos of TNG67, *Oscpn10a*, and *OsCPN10a* overexpression embryos. In *Oscpn10a* seeds, the ABA‐biosynthetic genes *OsZEP15*, *OsNCED3/4/5,* and the signaling factors *OsABI3/4/5*, *OsPYL4/10* were all significantly upregulated, whereas the catabolic genes *OsABA8ox1/2/3* were significantly downregulated (Figures 4A–C, S7). Ultra‐performance liquid chromatography analysis further confirmed that this transcriptional reprogramming was translated into a comparable ABA burst, detected in *Oscpn10a* seeds, similar to that of TNG67 during the early phase of accelerated aging (Figure 4D). In agreement with the expression pattern of ABA‐related genes, endogenous ABA in *Oscpn10a* embryos was also significantly higher than that of TNG67 after 6 h of imbibition (Figure 4E), whereas the *OsCPN10a* overexpression lines contained less ABA than TNG67 (Figure 4E), consistent with the decreased seed vigor phenotype in *Oscpn10a* mutants. The findings demonstrated that knockout of *OsCPN10a* tilts ABA homeostasis toward accumulation and sustained dormancy.

Since excessive ABA accumulation inhibits seed germination in plants (Ali et al., 2022), we assumed that treating *Oscpn10a* mutants with ABA might change the threshold for sensing or transducing ABA, resulting in a hormone hypersensitive phenotype. Indeed, 5 μM exogenous ABA suppressed germination in TNG67, *Oscpn10a* mutants, and *OsCPN10a* overexpression line seeds, but the inhibitory effect was significantly greater in the *Oscpn10a* mutants than in TNG67 (Figure 4G). Notably, 20 μM ABA treatment significantly reduced the GR of the *Oscpn10a* mutants were 54.55%–57.58% lower than those of TNG67 (Figure 4G). Correspondingly, root length and coleoptile length were repressed by the knockout of *OsCPN10a* in rice. For example, in *Oscpn10a* mutants, 5 μM ABA treatment significantly reduced root length by 79.12% (*Oscpn10a‐2*) and 71.38% (*Oscpn10a‐4*), and coleoptile length by 47.83% (*Oscpn10a‐2*) and 56.52% (*Oscpn10a‐4*), relative to TNG67 (Figure 4H, I). These results demonstrated that *OsCPN10a* modulates seed germination by ABA metabolism, suggesting its pivotal role in balancing ABA flux during seed germination in rice.

**Figure 5:**
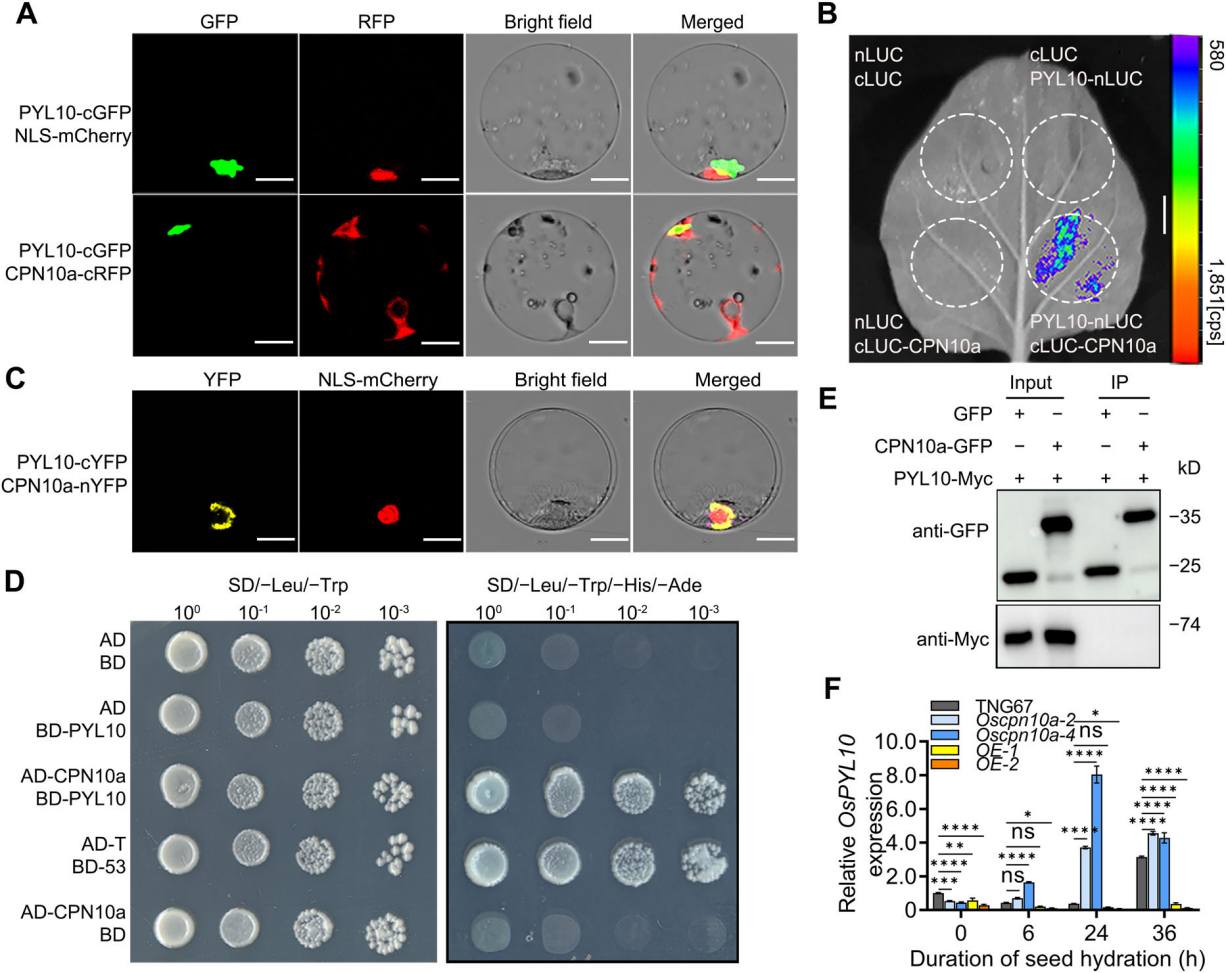
OsCPN10a interacts with OsPYL10 **(A)** Subcellular co‐localization of OsCPN10a and OsPYL10 proteins in the cytoplasm. Scale bars, 10 μm. **(B)** Split luciferase complementation assay of the interaction between OsCPN10a and OsPYL10 in *N. benthamiana* leaves. The luminescence intensity is expressed as counts per second (cps). Scale bars, 1 cm. **(C)** BiFC assay of the interaction between OsCPN10a and OsPYL10 in rice protoplasts. Scale bars, 10 μm. **(D)** Yeast two‐hybrid assay of the interaction between OsCPN10a and OsPYL10. **(E)** Co‐IP assay of the interaction between OsCPN10a and OsPYL10 *in vivo*. Total protein extracted from rice protoplasts co‐expressing OsPYL10‐Myc and OsCPN10a‐GFP, immunoprecipitated with GFP‐Trap beads. The precipitates were detected using anti‐GFP and anti‐Myc antibodies, respectively. Plus (+) and minus (−) signs indicate the presence or absence of the indicated protein in each group. **(F)** Relative expression of *OsPYL10* in *Oscpn10a* transgenic line embryos compared to wild‐type TNG67 embryos during seed germination. *OsACTIN150* was used as the internal control. Data are presented as means ± *SD* from three biologically independent replicates (*n* = 3; *P < 0.05, **P < 0.01, ***P < 0.001, ****P < 0.0001; Student's *t*‐test).

To further elucidate the seed germination and ABA metabolism regulation networks mediated by *OsCPN10a*, we performed an IP‐MS assay to identify OsCPN10a‐interacting protein candidates (Figure S8). Among the candidates was the ABA receptor OsPYL10 (Figure S9), prompting us to test a direct association. A subcellular co‐localization assay confirmed the co‐localization of these two proteins in the cytoplasm (Figure 5A). The results of luciferase complementation imaging (LUC) assay, bimolecular fluorescence complementation (BiFC), and yeast two‐hybrid (Y2H) assays confirmed an interaction between OsCPN10a and OsPYL10 (Figure 5B–D); while Co‐IP analysis failed to verify that the OsPYL10‐Myc protein interacted specifically with the OsCPN10a‐GFP protein (Figure 5E), implying the contact is transient or bridged by an intermediate. Consistent with an indirect regulatory mode, *OsPYL10* transcript levels were elevated in *Oscpn10a* embryos and repressed in *OsCPN10a* overexpression line embryos (Figure 5F), demonstrating that *OsCPN10a* negatively regulates *OsPYL10* transcription.

### OsCPN10a–OsCPN20–OsHSP60‐3B complex affects the interaction between OsPYL10 and OsABIL1

**Figure 6:**
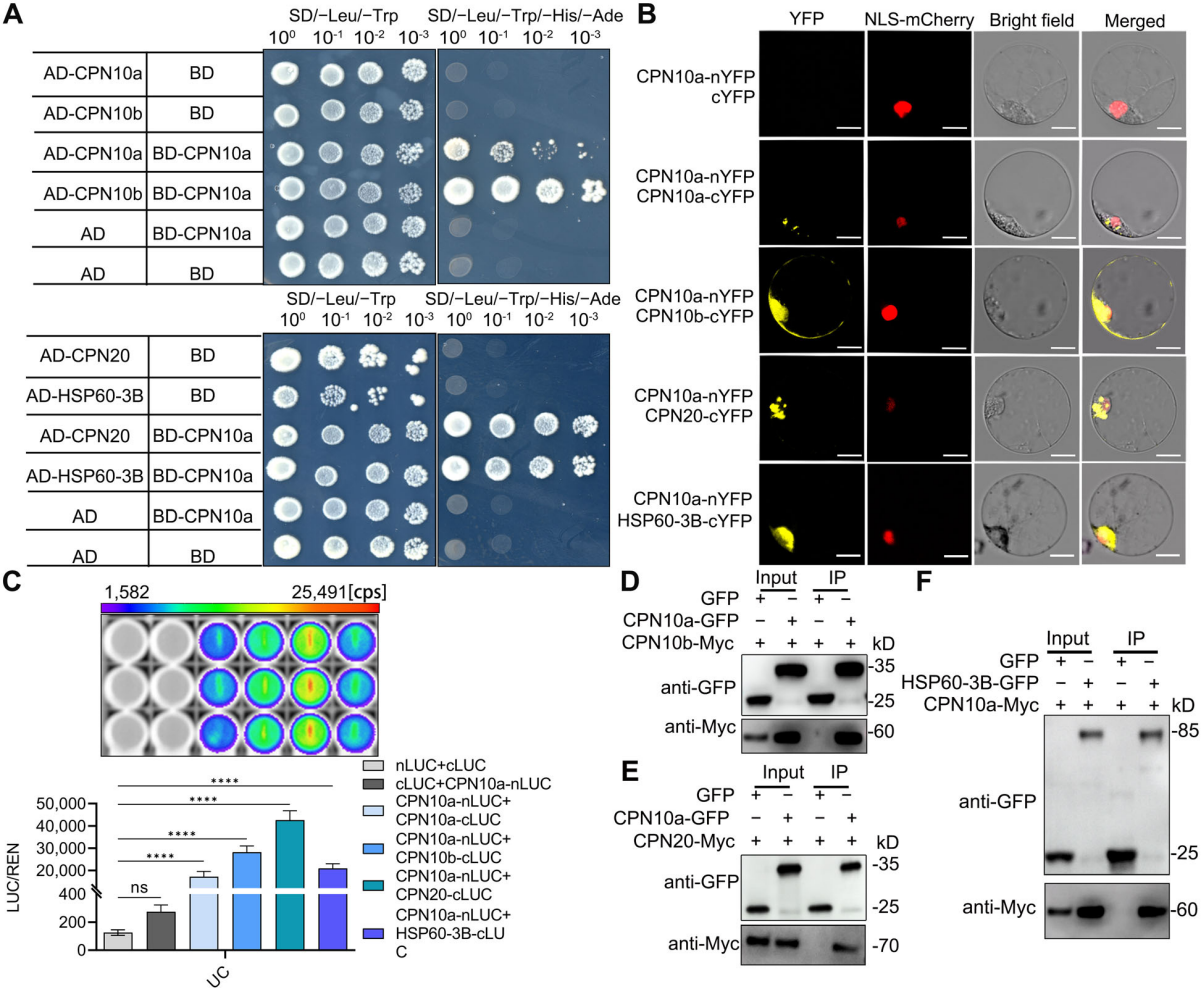
OsCPN10a interacts with OsCPN10b, OsCPN20, and OsHSP60‐3B **(A)** OsCPN10a interacts with OsCPN10b, OsCPN20, and OsHSP60‐3B in yeast two‐hybrid assays. **(B)** BiFC assay of the interaction between OsCPN10a and OsCPN10b, OsCPN20, and OsHSP60‐3B in rice protoplasts. Scale bars,10 μm. **(C)** LUC assay of the interaction between OsCPN10a and OsCPN10b, OsCPN20, and OsHSP60‐3B in rice protoplasts. Quantification of luminescence intensity measured as cps = counts per second (chemiluminescence value). Data are means ± *SD* from three biological replicates (*n* = 3; ****P < 0.0001; Student's *t*‐test). **(D**–**F)** Co‐IP assays confirm that OsCPN10a interacts with OsCPN10b **(D)**, OsCPN20 **(E)**, and OsHSP60‐3B **(F)** *in vivo*. Total protein was extracted from rice protoplasts co‐expressing OsCPN10a‐GFP and OsCPN10b‐Myc, OsCPN10a‐GFP and OsCPN20‐Myc, or OsCPN10a‐Myc and OsHSP60‐3B‐GFP and then immunoprecipitated with GFP‐Trap or Myc‐Trap beads. The precipitates were detected using anti‐GFP and anti‐Myc antibodies, respectively.

Having established that OsCPN10a indirectly curbs OsPYL10 transcription to fine‐tune ABA signaling, we next asked whether its cytoplasmic chaperone network also extends to heat‐shock proteins, the canonical partners required for stress proteostasis and seed longevity. Notably, IP‐MS screening identified several HSP family proteins, including OsCPN10b, OsCPN20, and OsHSP60‐3B, as candidate interacting partners of OsCPN10a (Figure S9). To explore the functional relevance of these interactions, we performed a series of *in vitro* and *in vivo* experiments. RT‐qPCR revealed that *OsCPN10b* transcription was elevated in *Oscpn10a* mutant embryos but suppressed in *OsCPN10a* overexpression lines (Figure S10A). In contrast, *OsCPN20* was significantly upregulated in both *Oscpn10a* mutants and OsCPN10a overexpression embryos, whereas *OsHSP60‐3B* was distinctly downregulated (Figure S10B, C). Co‐localization analysis revealed that OsCPN10a co‐localizes with OsCPN10b and OsCPN20 exclusively in the cytoplasm, whereas it co‐localizes with OsHSP60‐3B within the nucleus (Figure S10D). To validate this observation, we performed Y2H, BiFC, LUC, and Co‐IP assays, which confirmed that OsCPN10a interacts directly with these HSP proteins *in vivo* (Figure 6A–F). This data also indicated that OsCPN10a can function as a heterodimer.

Given that OsCPN10a was shown to form homo‐ and heterodimers with other HSP family members, we further investigated whether its homodimerization is regulated by interacting proteins. To this end, Co‐IP assays were performed in rice protoplasts transiently expressing OsCPN20‐GFP, OsCPN10a‐Myc, and OsCPN10a‐HA to analyze the effect of varying OsCPN20 concentrations on OsCPN10a homodimerization. Intriguingly, stable OsCPN10a homodimers were detected at low OsCPN20 concentrations, whereas the level of OsCPN10a‐Myc/HA homodimers decreased significantly with increasing OsCPN20‐Myc concentrations (Figure S11A), indicating that OsCPN20 may interfere with OsCPN10a homodimerization. Although the Y2H assay indicated that OsCPN20 binds OsHSP60‐3B (Figure 6A), the Co‐IP experiments failed to detect the binding between OsCPN20‐GFP and OsHSP60‐3B‐Myc (Figure S11B). Transient co‐expression in rice protoplasts showed that when OsCPN20‐GFP and OsHSP60‐3B‐GFP were co‐expressed, only the OsHSP60‐3B‐GFP band was detectable, with OsCPN20‐GFP remaining undetectable (Figure S11B, C). However, in the presence of OsCPN10a‐Myc, prominent bands for OsCPN20‐GFP, OsHSP60‐3B‐GFP, and their co‐expressed counterparts were consistently detected (Figure S11C). Collectively, these results demonstrated that OsCPN20 is destabilized or degraded unless protected by assembly into the OsCPN10a multimeric complex.

**Figure 7:**
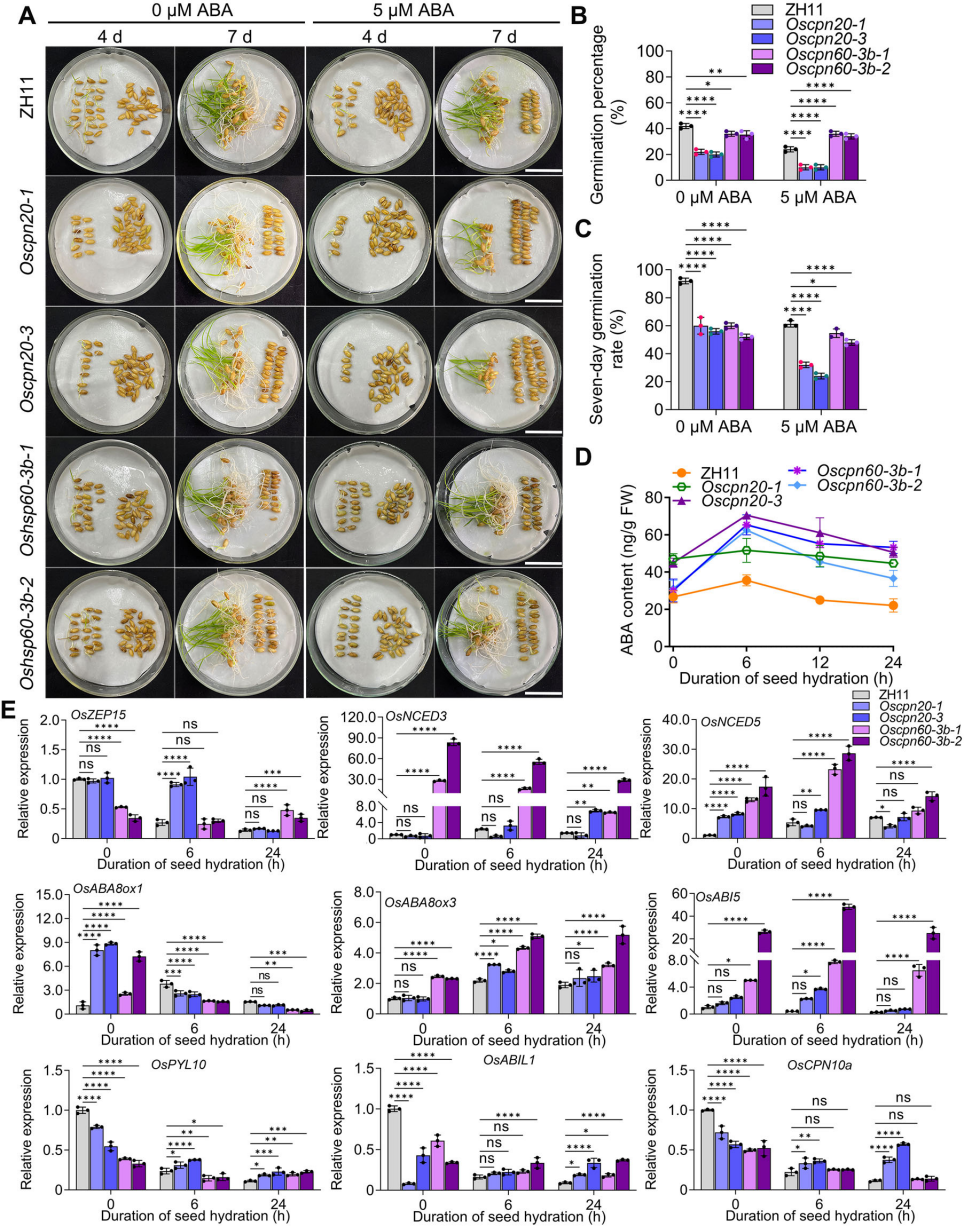
Genetic analyses of OsCPN20 and OsHSP60 **(A)** Representative images of wild‐type (ZH11), *Oscpn20* mutant, and *Oshsp60‐3b* mutant seed germination over 4 and 7 d under 0 or 5 μM ABA treatment. Scale bar, 1 cm. **(B**, **C)** The 4‐d germination rate and 7‐d germination rate of ZH11, *Oscpn20* mutants, and *Oshsp60‐3b* mutants in response to ABA treatment. **(D)** ABA contents in germinated embryos of ZH11, *Oscpn20* mutant, and *Oshsp60‐3b* mutant seeds. **(E)** Relative expression of ABA biosynthesis genes (*OsZEP15* and *OsNCED3/5*), ABA degradation genes (*OsABA8ox1/3*), ABA signaling genes (*OsABI5*, *OsPYL10*, and *OsABIL1*), and *OsCPN10a* in germinated embryos of ZH11, *Oscpn20* mutant, and *Oshsp60‐3b* mutant seeds, respectively. Relative expression is represented as the fold‐change relative to expression in ZH11 0 h, 6 h, and 24 h after imbibition. *OsACTIN150* was used as the internal control (*n* = 3). In **(B**–**E)**, data are given as means ± *SD* from three biological replicates (*n* = 3). Student's *t*‐test was used to generate the *P‐*values: *P < 0.05, **P < 0.01, ***P < 0.001, ****P < 0.0001.

Since OsCPN10a interacts with OsCPN20 and OsHSP60‐3B, we hypothesized that these co‐chaperones might modulate seed vigor by influencing ABA metabolism during germination. Consistent with this suggestion, RT‐qPCR results showed that the expression level of *OsCPN20* and *OsHSP60‐3B* rose gradually within 24 h and then exhibited a downward trend after exogenous ABA treatment (Figure S11D). To elucidate the effects of *OsCPN20* and *OsHSP60‐3B* on seed germination, we generated knockout mutants of two genes in the ZH11 background using CRISPR/Cas9 technology. Sequencing confirmed three homozygous *Oscpn20* mutant lines (*Oscpn20‐1*, *Oscpn20‐2*, and *Oscpn20‐3*) and two homozygous *Oshsp60‐3b* mutant lines (*Oshsp60‐3b‐1* and *Oshsp60‐3b‐2*) (Figures S12, S13). Germination assays revealed that both the 4‐d GP (36.67% and 46.67% for the *Oscpn20‐1* and *Oscpn20‐3* mutants vs. 56.75% for the wild‐type ZH11) and the 7‐d GR (56.67% and 46.67% for the *Oscpn20‐1* and *Oscpn20‐3* mutants vs. 88.75% for ZH11) were significantly lower in the *Oscpn20* mutants (Figure 7A–C). Similarly, phenotypic analyses indicated that the 4‐d GP and 7‐d GR of the *Oshsp60‐3b* mutants were significantly lower than those of ZH11 (Figure 7A–C). In addition, when treated with 5 μM ABA, the 4‐d GP and 7‐d GR of the *Oscpn20* mutants were significantly lower than those without ABA treatment (Figure 7A–C). Notably, we observed a significant accumulation of ABA in *Oscpn20* and *Oshsp60‐3b* mutants during the seed germination process (Figure 7D), which may account for the observed reduction of germination.

We next analyzed the transcript levels of key ABA pathway genes in germinating seed embryos of the *Oscpn20* and *Oshsp60‐3b* mutants. Compared to ZH11, *Oscpn20* and *Oshsp60‐3b* mutant embryos exhibited significantly upregulated expression of *OsZEP15*, *OsNCED3/5*, *OsABA8ox1/3*, and *OsABI5* (Figure 7E). In addition, *OsPYL10* and *OsABIL1* were significantly upregulated in these mutants after 6 h and 24 h of imbibition (Figure 7E). These data indicated that *OsCPN20* and *OsHSP60‐3B* are both involved in regulating ABA signal metabolism during seed germination.

**Figure 8:**
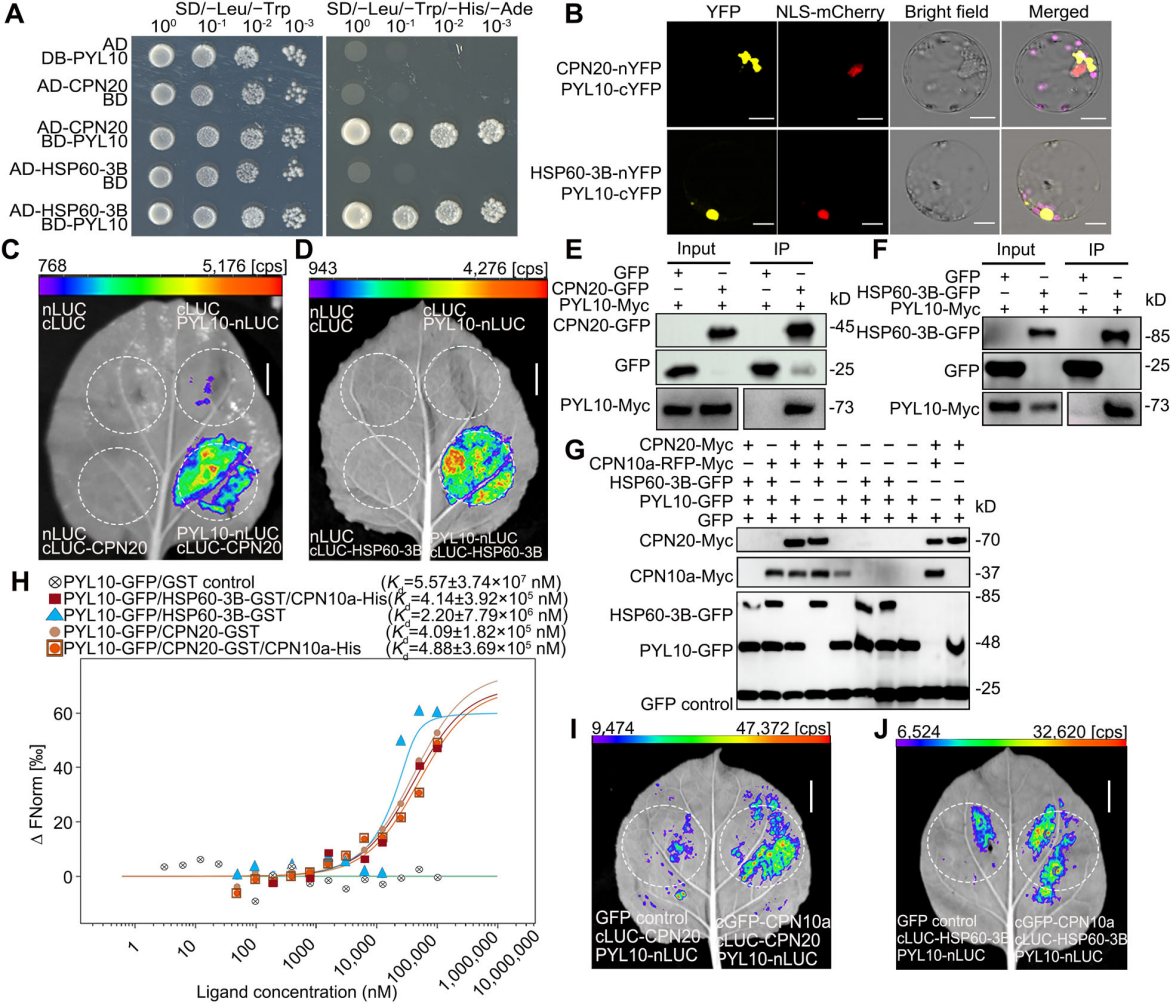
OsCPN10a stabilizes the chaperone complex and enhances its interaction with OsPYL10 **(A)** Yeast two‐hybrid assay of the interaction of OsPYL10 with OsCPN20 and OsHSP60‐3B. **(B)** BiFC assay of the interaction of OsPYL10 with OsCPN20 and OsHSP60‐3B in rice protoplasts. Scale bar, 10 μm. **(C**, **D)** Split luciferase complementation assay of the interaction between OsPYL10 and OsCPN20 and OsHSP60‐3B in *N. benthamiana* leaves. Scale bars, 1 cm. **(E**, **F)** Co‐IP assay of the interaction of OsPYL10 with OsCPN20 and OsHSP60‐3B in rice protoplasts. **(G)** Immunoblot analysis of OsCPN20, OsHSP60‐3B, and OsPYL10 protein levels in rice protoplasts co‐expressing OsPYL10‐GFP with OsCPN20‐Myc and OsHSP60‐3B‐GFP in the presence or absence of OsCPN10a‐Myc. Proteins were detected with anti‐GFP and anti‐Myc antibodies. GFP empty vector served as a loading control. **(H)** MST analysis of the binding affinity between purified OsPYL10‐GFP and OsCPN20‐GST or OsHSP60‐3B‐GST in the presence or absence of purified OsCPN10a‐His. OsPYL10‐GFP was purified from rice protoplasts using GFP‐Trap beads. OsCPN10a‐His, OsCPN20‐GST, and OsHSP60‐3B‐GST were purified from *Escherichia coli Rosetta* (*DE3*) using His‐Trap or GST‐Trap beads, respectively. The normalized fluorescence change (ΔFNorm [‰]) was plotted against ligand concentration. Data represent means ± *SD* from three independent experiments (biological replicates), with 16 serial dilutions (technical replicates) per experiment. Representative binding curves are shown. **(I**, **J)** LUC assays showed that OsCPN10a affects the interactions of both OsCPN20–OsPYL10 and OsHSP60–OsPYL10 in a transient expression assay in *N. benthamiana* leaves. Scale bars, 1 cm.

Given that OsCPN10a, OsCPN20, and OsHSP60‐3B all positively regulate seed germination, we hypothesized that the OsCPN10a–OsCPN20–OsHSP60‐3B complex modulates seed vigor by engaging with the ABA signaling pathway, possibly through functional interaction with OsPYL10. To test this, we next assessed the potential interaction between the OsCPN20, OsHSP60‐3B, and OsPYL10. We used a Y2H assay to confirm that OsCPN20 and OsHSP60‐3B interact with OsPYL10 *in vivo* (Figure 8A). Moreover, the results of BiFC, LUC, and Co‐IP assays showed that OsPYL10 interacts with OsCPN20 and OsHSP60‐3B (Figure 8B–D, F). Western blot (WB) assays further demonstrated that none of the OsCPN10a, OsCPN20, and OsHSP60‐3B affect the stability altered OsPYL10 abundance *in vivo* (Figure 8G). This result was further confirmed by MST assay using rice protoplasts transiently expressing OsPYL10‐GFP and prokaryotes (*Rosetta*) expressing proteins OsCPN10a‐His, OsCPN20‐GST, and OsHSP60‐3B‐GST. OsPYL10 was shown to interact with both OsCPN20 and OsHSP60‐3B *in vitro*, with OsCPN10a promoting these interactions (Figure 8H). However, LUC assay revealed that OsCPN10a promotes interaction between OsCPN20–OsPYL10 and OsHSP60‐3B–OsPYL10 (Figure 8I, J). These results indicated that OsCPN10a, OsCPN20, and OsHSP60‐3B might form a complex to exert their functions.

**Figure 9:**
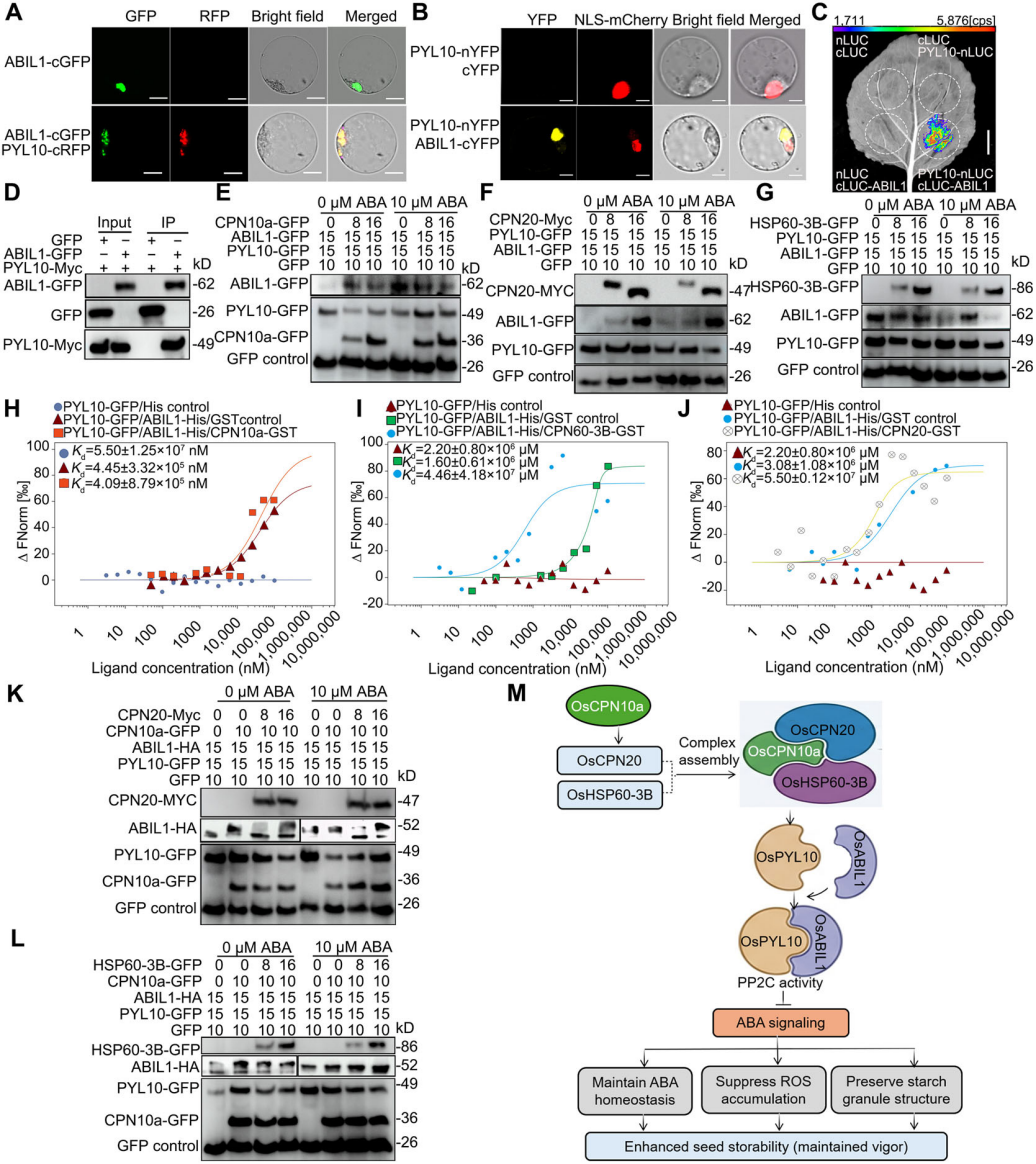
The OsCPN10a–OsCPN20–OsHSP60‐3B complex stabilizes the association between OsPYL10 and OsABIL1 **(A)** Subcellular co‐localization of OsPYL10 and OsABIL1 in rice protoplasts. Scale bar, 10 μm. **(B)** BiFC assay of the association between OsPYL10 and OsABIL1 in rice protoplasts. Scale bar, 10 μm. **(C)** Split luciferase complementation assay of the association between OsPYL10 and OsABIL1 in *N. benthamiana* leaves. Scale bars, 1 cm. cps, counts per second (chemiluminescence value). **(D)** Co‐IP assay of proteins extracted from rice protoplasts co‐expressing OsPYL10‐Myc and OsABIL1‐GFP. Total protein was immunoprecipitated with GFP‐Trap beads and detected using anti‐GFP and anti‐Myc antibodies. **(E**–**G)** Immunoblot analysis of OsPYL10 and OsABIL1 protein levels in rice protoplasts co‐expressing OsPYL10‐GFP and OsABIL1‐GFP with or without **(E)** OsCPN10a‐GFP, **(F)** OsCPN20‐Myc, or **(G)** OsHSP60‐3B‐GFP. Proteins were detected with anti‐GFP and anti‐Myc antibodies. GFP empty vector served as a loading control. **(H**–**J)** MST analysis of the binding affinity between purified OsPYL10‐GFP and OsABIL1‐His in the presence or absence of **(H)** OsCPN10a‐GST, **(I)** OsCPN20‐GST, or **(J)** OsHSP60‐3B‐GST. OsPYL10‐GFP was purified from rice protoplasts using GFP‐Trap beads. OsABIL1‐His, OsCPN10a‐GST, OsCPN20‐GST, and OsHSP60‐3B‐GST were purified from *Escherichia coli Rosetta* (*DE3*) using His‐Trap or GST‐Trap beads, respectively. The normalized fluorescence change (ΔFNorm [‰]) was plotted against ligand concentration. Data represent means ± *SD* from three independent experiments (biological replicates), with 16 serial dilutions (technical replicates) per experiment. Representative binding curves are shown. **(K)** Immunoblot analysis of OsPYL10 and OsABIL1 protein levels in rice protoplasts co‐expressing OsPYL10‐GFP and OsABIL1‐HA with or without simultaneous co‐expression of OsCPN10a‐GFP and OsCPN20‐GFP. Proteins were detected with anti‐GFP, anti‐HA, and anti‐Myc antibodies. GFP empty vector served as a loading control. **(L)** Immunoblot analysis of OsPYL10 and OsABIL1 protein levels in rice protoplasts co‐expressing OsPYL10‐GFP and OsABIL1‐HA with or without simultaneous co‐expression of OsCPN10a‐GFP and OsHSP60‐3B‐GFP. Proteins were detected with anti‐GFP and anti‐HA antibodies. GFP empty vector served as a loading control. **(M)** Model of the OsCPN10a‐mediated chaperone module regulating ABA signaling during high‐temperature storage. During high‐temperature storage‐induced aging, the OsCPN10–OsCPN20–OsHSP60‐3B trimeric complex interacts with OsPYL10, promoting the dissociation of dimeric PYR/PYL/RCAR receptors and their high‐affinity association with the PP2C phosphatase OsABIL1. Sequestration of OsABIL1 prevents the autophosphorylation and activation of downstream SnRK2 kinases, thereby blocking ABA signaling and suppressing seed aging. Suppression of ABA signaling further inhibits ROS accumulation and amyloplast starch granule disintegration, ultimately delaying seed aging and maintaining seed viability.

PYL10 is an ABA receptor that inhibits protein phosphatase 2C (PP2C) activity in *A. thaliana* (Sun et al., 2012). This inhibition prevents PP2C from dephosphorylating and inactivating SnRK2s (sucrose non‐fermenting 1‐related protein kinase‐2s), thereby facilitating the activation of ABA signaling pathways and downstream stress responses. Subcellular colocalization and BiFC assays revealed that OsABIL1 localized exclusively in the nucleus, whereas OsPYL10 localized in the cytoplasm. Upon interaction, OsABIL1 is recruited to the cytoplasm, where the two proteins form a stable OsPYL10–OsABIL1 complex (Figure 9A, B). In addition, LUC and Co‐IP assays further demonstrated that OsPYL10 and OsABIL1 form a direct protein complex *in vivo* (Figure 9C, D). These results indicated that OsPYL10 directly interacts with OsABIL1 *in vivo*.

Previous studies have reported that PP2C phosphatase inhibition by PYL10 is ABA‐dependent (Li et al., 2015). As the OsCPN10a–OsCPN20–OsHSP60‐3B complex is associated with OsPYL10 to regulate ABA signaling, we speculated that these co‐chaperones may stabilize the interaction between OsPYL10 and OsABIL1. To test this possibility, we conducted WB analysis of rice protoplasts and found that when OsCPN10a, OsCPN20, or OsHSP60‐3B was expressed, the abundance of OsABIL1 increased in a dose‐dependent manner, while the content of OsPYL10 remained unchanged *in vivo* (Figure 9E–G). MST analysis corroborated this stabilizing effect that OsABIL1‐His bound OsPYL10‐GFP with high‐affinity interaction, and the inclusion of OsCPN10a‐GST further enhanced the interaction (Figure 9H). Likewise, addition of OsCPN20 or OsHSP60‐3B enhanced the thermal stability of the OsABIL1–OsPYL10 complex *in vitro* (Figure 9I, J), demonstrating that the entire OsCPN10a module actively stabilizes the ABA receptor complex. In the absence of both OsCPN10a and ABA, OsHSP60‐3B alone could not fully prevent OsABIL1 degradation but still stabilized OsPYL10 (Figure 9G). Conversely, when OsCPN10a was present, it enabled OsCPN20 or OsHSP60‐3B to synergistically protect both OsPYL10 and OsABIL1 under ABA treatment (Figure 9K, L). Thus, the OsCPN10a–OsCPN20–OsHSP60‐3B chaperone module fine‐tunes the OsPYL10–OsABIL1 interaction to modulate ABA signaling, ultimately controlling ABA accumulation and seed germination, as summarized in our working model (Figure 9M).

## DISCUSSION

Enhanced seed storability and precise regulation of seed vigor act synergistically to enable plants to preserve genetic potential, optimize germination timing, and ensure seedling survival under diverse environmental conditions (Zhou et al., 2019; Reed et al., 2022). It is of vital importance to generate superior seed storability crop varieties, especially those that can sustain seed vigor during prolonged storage. Currently, very few genes capable of enhancing storage tolerance in rice have been reported, but here, we show that *OsCPN10a* can improve seed storability.

In this study, OsCPN10a was genetically and molecularly identified as a positive regulator of seed storability by maintaining the stability of starch metabolic enzymes, promoting antioxidant activities, and participating in ABA signal transduction. We also demonstrated that OsCPN10a assembles a hetero‐oligomeric OsCPN10a–OsCPN20–OsHSP60‐3B chaperone complex that stabilizes the OsPYL10–OsABIL1 module, thereby regulating ABA signaling and seed vigor. Our findings redefine the functional scope of co‐chaperonins in plants. We establish OsCPN10a not merely as an accessory folding factor but as a central regulator that coordinates protein homeostasis, redox balance, and hormone signaling to optimize seed performance. This molecular framework offers a direct genetic target for enhancing rice seed longevity and germination performance.

### OsCPN10a positively regulates seed storability by protecting starch reserves and maintaining redox homeostasis

Under aging stress, the chaperone system serves as a critical line of defense for maintaining cellular homeostasis (Kim et al., 2013; Freilich et al., 2018). This study demonstrates that recombinant OsCPN10a protein effectively suppresses heat‐induced MDH aggregation *in vitro* (Figure 3F), exhibiting typical chaperone activity. Further investigation reveals that OsCPN10a localizes to the nucleus, endoplasmic reticulum, and peroxisomes (Figure 1), suggesting its potential involvement in stress defense through multi‐organelle coordination. Genetic evidence demonstrates that *OsCPN10a* is a critical positive regulator of seed storability, as *Oscpn10a* mutants exhibit drastically reduced germinability under both artificial and natural aging, whereas *OsCPN10a* overexpression enhances stress tolerance (Figures 1, S4).

During accelerated aging, seeds undergo severe dehydration accompanied by ROS bursts, which disrupt the architecture of starch and protein within the endosperm, thereby impairing energy supply for germination (Han et al., 2022; Wu et al., 2022a; He et al., 2024). Beyond its canonical role in protein folding, OsCPN10a actively regulates cellular redox homeostasis to break the vicious cycle of seed aging. This study demonstrates that *OsCPN10a* deficiency significantly exacerbates both ROS accumulation and starch deterioration during seed aging (Figure 2). Specifically, *Oscpn10a‐4* mutants exhibit 2.8‐fold higher H_2_O_2_ levels and 1.4‐fold elevated MDA content compared to WT at 14 d (Figure 2B, C), concomitant with severely compromised activities of α‐amylases, SOD, POD, and CAT (Figure 3D, E, G, I). These results suggest that OsCPN10a may directly stabilize key starch metabolic enzymes (e.g., α‐amylases, branching enzymes) to prevent metabolic disorganization, or indirectly ensure proper folding and sustained activity of antioxidant enzymes to maintain redox homeostasis. This protective function is evolutionarily conserved with other small HSPs such as *OsHSP18.2*, *AtHSP17.4*, and *HaHSP17.6* (Kaur et al., 2015; Wu et al., 2022b; Chen, 2023).

Notably, the role of the interacting protein OsHSP60‐3B in starch protection via FLOURY ENDOSPERM6 (FLO6) stabilization is well‐established (Lin et al., 2023). Lin et al. (2023) demonstrated that OsHSP60‐3B interacts directly with FLO6, a key starch‐binding protein, to regulate starch granule biogenesis in rice pollen under high temperature. Importantly, the *Oshsp60‐3b* mutant exhibited not only defective starch accumulation but also elevated ROS levels and increased cell death in anthers, establishing a direct link between HSP60‐3B function and redox homeostasis. Building upon this evidence, we propose that the OsCPN10a–OsCPN20–OsHSP60‐3B complex identified in our study extends this dual protective mechanism to seed storage contexts, a hypothesis that warrants further mechanistic investigation.

### OsCPN10a non‐canonically modulates ABA signaling to coordinate seed aging and germination

ABA is a key phytohormone involved in seed maturation, dormancy, and stress responses (Diaz‐Martin et al., 2005; Ali et al., 2022). While ABA generally delays seed aging by suppressing ROS accumulation, prolonged storage often leads to declining ABA levels, elevated H_2_O_2_, and consequent loss of vigor (Chen et al., 2019; Zheng et al., 2024). This study reveals that *OsCPN10a* modulates this dynamic balance. In *Oscpn10a* mutants, ABA levels exhibit a transient compensatory increase during early aging but subsequently decline, leading to uncontrolled ROS accumulation and accelerated viability loss (Figures 2B, 4D). Conversely, *OsCPN10a* overexpressing lines maintained elevated ABA levels and decreased H_2_O_2_ levels throughout 14 d of artificial aging (Figures 2B, 4D). These results suggest that *OsCPN10a* enhances aging tolerance by modulation of ABA homeostasis.

RT‐qPCR analyses further provide mechanistic insights into this regulatory mode. ABA biosynthetic genes are significantly enriched in *Oscpn10a* mutants versus TNG67 (Figure S7A), while key signaling regulators (OsABI3/4/5) are hyperactivated in *Oscpn10a* embryos but suppressed in overexpression lines (Figures 4, S7B). The resulting ABA hypersensitivity of *Oscpn10a* seeds confirms that *OsCPN10a* fine‐tunes ABA signaling to optimize germination timing (Figure 4). Collectively, these findings expand the functional scope of molecular chaperones from protein homeostasis to environmental signal integration, revealing that *OsCPN10a* optimizes ABA signaling efficiency rather than merely suppressing the pathway. This study provides mechanistic insights into ABA–H_2_O_2_ crosstalk governing seed aging and vigor maintenance, offering a potential strategy for enhancing seed storability through targeted modulation of chaperone‐mediated hormone signaling.

Notably, HSPs play specific roles in ABA signaling, yet their mechanisms vary across species. In *A. thaliana*, AtCPN20 operates independently in chloroplasts, interacting upstream with the ABA receptor CHLH/ABAR to regulate WRKY40‐mediated signaling (Zhang et al., 2014), whereas AtCPN10(1) acts solely as an AtCPN20‐dependent chaperone without direct involvement in ABA transduction (Vitlin Gruber et al., 2014). This study reveals that rice has evolved into a distinct regulatory architecture. OsCPN10a nucleates the assembly of a cytoplasmic hetero‐oligomeric complex with OsCPN20 and OsHSP60‐3B, creating functional microdomains that facilitate oligomerization (Figure 6), representing a significant evolutionary divergence from the Arabidopsis model. Unlike AtCPN20, which operates upstream of CHLH/ABAR, the OsCPN10a–OsCPN20–OsHSP60‐3B complex acts directly at the receptor–phosphatase interface. This configuration enables rapid germination under favorable conditions while maintaining aging tolerance during storage, reflecting a sophisticated regulatory network evolved in monocots to adapt to specific environmental constraints.

### OsCPN10a bridges protein homeostasis and hormone signaling through complex‐mediated receptor modulation

In rice, the core ABA receptor PYLs act as the on switch for ABA signaling (Wang et al., 2024). Upon ABA binding, PYLs undergo conformational changes that enable interaction with PP2C phosphatases, inhibiting their activity and activating downstream responses (Sun et al., 2012). Previous studies established that AtPYL10 interacts with AtABI1 *in vitro* in an ABA‐independent manner (Hao et al., 2011; Li et al., 2015). Our findings extend this to rice, demonstrating that OsABIL1 physically interacts with OsPYL10 (Figure 9), supporting a conserved yet flexible interaction network across species.

Mechanistically, OsCPN10a does not bind OsPYL10 directly but nucleates the assembly of a cytoplasmic hetero‐oligomeric complex with OsCPN20 and OsHSP60‐3B. Both OsCPN20 and OsHSP60‐3B interact directly with the core ABA receptor OsPYL10 (Figure 8), enabling the chaperone module to dock onto the receptor and stabilize its interaction with the phosphatase OsABIL1, thereby promoting the “off” state of ABA signaling (Figure 9M). Genetic evidence confirms that OsCPN20 and OsHSP60‐3B function downstream of OsCPN10a, as their knockout mutants phenocopy the ABA hypersensitivity and germination inhibition of *Oscpn10a* seeds (Figure 7). While our biochemical data demonstrate physical interactions between the chaperone complex and OsPYL10, formal demonstration that this complex stabilizes the OsPYL10–OsABIL1 interface *in planta* awaits future genetic studies. Specifically, generating *Oscpn10a*/*Ospyl10* and *Oscpn10a*/*Osabil1* double mutants would allow rigorous testing of whether loss of ABA receptor or phosphatase suppresses the hypersensitive phenotype of *Oscpn10a*, thereby providing definitive *in vivo* validation of the proposed mechanism.

In summary, this study reveals a dual‐function mechanism by which the OsCPN10a–OsCPN20–OsHSP60‐3B complex regulates seed storability. This module safeguards starch reserves and redox homeostasis to enhance storability, while modulating ABA signaling through stabilization of the OsPYL10–OsABIL1 interface to optimize germination timing. By bridging protein homeostasis with hormone signal integration, OsCPN10a serves as a molecular switch balancing seed longevity and germination performance, offering a valuable genetic resource for improving seed quality in rice molecular breeding.

## MATERIALS AND METHODS

### Plant materials and growth conditions

Fourteen elite rice varieties, including Fuxiangzhan (FXZ), Yuejingsimiao 2, H603, TNG67, Nipponbare (NIP), Minghui 86, 676, 93‐11, Qingjingzhan, Huazhan, Yujingyouzhan, Yujingshimiao, Yuzhenxiang, and Yunhui 290, were evaluated for seed aging in this study. Stable transgenic lines of *OsCPN10a* overexpression lines (*OE‐1* and *OE‐2*), CRISPR–Cas9‐edited mutants (*Oscpn10a*, *Oscpn20*, and *Oshsp60*‐*3b*), and *proUbi: OsCPN10a* were used for gene expression and phenotype analysis. All the plants were grown at two experimental stations of the Rice Research Institute, Fujian Academy of Agricultural Sciences in Fuzhou (Fujian Province) and Lingshui (Hainan Province), respectively, under natural growth conditions. For seed germination analysis, seeds were germinated in a growth chamber at 30°C and 60% relative humidity in a 14‐h light/12‐h dark cycle for 7 d.

### 2,3,5‐triphenyltetrazolium chloride assay

Rice seeds were de‐hulled and pre‐hydrated in sterile water for 3 h, then incubated in 1% (w/v) TTC (Biosharp, Cat. BS095) solution (pH 7.0 phosphate buffer) at 30°C in darkness for 2 h. After rinsing three times, embryos showing uniform deep‐red staining were scored as viable. The samples were analyzed using a stereomicroscope (SMZ800N, Nikon).

### RNA isolation and reverse transcription quantitative real‐time PCR (RT‐qPCR) analysis

Total RNA was extracted from rice tissues using the TRIzol Reagent (Invitrogen, Carlsbad, CA, USA) according to the manufacturer's protocol. Complementary cDNA was synthesized with 1 μg RNA using ReverTra Ace® qPCR RT Master Mix (TOYOBO Biotech, Osaka, Japan). RT‐qPCR was performed using a Roche LightCyclerr 480II instrument (Roche, Jena, Germany) and the FastStart Universal SYBR Green Master (Rox) (Roche, Shanghai, China). The rice *actin150* gene was used as the internal control, and the relative expression levels were calculated using the 2^−ΔΔCT^ method. The primers used in this study are listed in Table S1.

### Plasmid construction

The *Oscpn10a‐1*, *Oscpn10a‐2*, *Oscpn10a‐3*, and *Oscpn10a‐4* mutants (TNG67 background) *and Oscpn20‐1*, *Oscpn20‐2*, *Oscpn20‐3*, *Oshsp60‐3b‐1*, *Oshsp60‐3b‐2* mutants (ZH11 background) were generated using the CRISPR–Cas9 system (Wang et al., 2023). gRNA constructs required for CRISPR/Cas9‐mediated generation of these gene alleles were designed as Wei et al. described (Wei et al., 2024). The promoters of *OsCPN10a* were first amplified from TNG67 genomic DNA templates using Phanta Max Super‐Fidelity DNA Polymerase (Vazyme, Nanjing, China), fused with the *β*‐glucuronidase (GUS) gene, and ligated into pRHE vectors to generate the pRHE*‐proOsCPN10a::*GUS vector. To achieve *OsCPN10a* overexpression, the full‐length coding sequences (CDS) of *OsCPN10a* were amplified from TNG67 cDNA templates without stop codons and cloned into the pRHV‐cGFP vector with the maize *Ubiquitin1* (*Ubi*) promoter to generate the pRHV‐*proUbi::OsCPN10a‐GFP* vector. pRHE*‐proOsCPN10a::*GUS and pRHV‐*proUbi::OsCPN10a‐GFP* vector were transformed into TNG67 by *A. tumefaciens*‐mediated transformation. The CDS of *OsCPN10b*, *OsHSP60‐3B*, *OsCPN10a*, *OsPYL10*, *OsCPN20*, and *OsABIL1* were ligated into pRTV‐cGFP or pRTV‐cRFP vectors. For Y2H assays, *OsCPN10b*, *OsCPN20*, *OsHSP60‐3B*, and *OsABIL1* were then inserted into pGADT7 vectors, while *OsPYL10* and *OsCPN10a* were also ligated into pGBKT7 and pGADT7 vectors. For BiFC assays, *OsCPN10b*, *OsABIL1*, and *OsHSP60‐3B* were then inserted into pRTV‐nVN (nYFP) vectors, while *OsCPN10a*, *OsPYL10*, and *OsCPN20* were also ligated into pRTV‐nVC (cYFP) and pRTV‐nVN (nYFP) vectors. For Co‐IP assays, *OsCPN10a*, *OsHSP60‐3B*, *OsABIL1*, *OsPYL10*, and *OsCPN20* were individually inserted into pRTV‐cGFP, pRTV‐cRFP‐Myc, nFy‐C‐MKK4‐Myc, or pRTV‐cRFP‐HA vectors to generate the corresponding epitope‐tagged fusions. For the MST assays, the full‐length CDSs of *OsCPN10a*, *OsCPN20*, and *OsHSP60‐3B* genes were inserted into the pGEX‐6P vector to yield N‐terminal GST fusions, whereas the CDSs of *OsABIL1* and *OsPYL10* genes were cloned into pET‐28a‐c (+) (N‐terminal 6× His) with His tag and pRTV‐cGFP (C‐terminal GFP), respectively.

### Homozygous *OsCPN10a* overexpression (OE) lines analysis

T_0_ plants were selected on 50 mg L^−1^ hygromycin and self‐pollinated; T_1_ seeds exhibiting a 3:1 resistant: Sensitive segregation on hygromycin (χ² test, *P* > 0.05) were considered single‐locus insertions. Resistant T_1_ individuals were advanced to T_2_, and lines that showed 100% hygromycin resistance (no segregation) were pre‐screened as putative homozygotes. Then, the copy number of the putative homozygotes was determined by RT‐qPCR using conum‐sfu2af‐F/R and conum‐HYG‐F/R (Liao et al., 2025). The pUC plasmid containing an *OsCPN10a* gene fragment was used to generate a standard curve.

### Evaluation of seed germination and artificial aging treatment

Seed germination was conducted as previously described (He et al., 2022). Briefly, approximately 50 seeds per replicate of each genotype were imbibed in a 90‐mm petri dish covered with two filter papers. Ten milliliters of distilled water were added, and the dishes were placed in a dark incubator (28°C, 12 h light) for 7 d. To test germination after breaking dormancy, germination was assayed following 48 h of chemical dormancy, released with 0.5 mM GA_3_ at 30°C. For ABA treatments, distilled water was replaced with solutions containing 0 and 5 μM ABA, respectively. Germination was considered to have occurred when the shoot length was half of the seed length, or the radicle length was the full length of the seed. Three biological replicates were performed. GP, GR, and GI were calculated as follows: GP (%) = (Number of seeds germinated in 4 d after 24 h of imbibition/Total number of seeds) × 100; GR (%) = (Number of seeds germinated in 7 d after 24 h of imbibition /Total number of seeds) × 100; and GI = ∑(Gt/t), where Gt represents the number of germinated seeds on a specific day and t is the total number of days (Liao et al., 2025). To carry out the artificial aging treatment, 50 seeds per replicate of each genotype were incubated in a ventilated heating chamber (Binder, Tuttlingen, Germany) for durations of 0, 7, 14, or 21 d under conditions of 42°C and 88% relative humidity. After artificial aging, seed germination was calculated as above.

### Histochemical staining

To assess the promoter activity of *proCPN10a*, GUS staining was performed on various organs from *proOsCPN10a*:*GUS* transgenic plants, using a GUS staining solution kit (G3061, Solarbio, Beijing, China) at 37°C for 4–24 h in darkness according to the manufacturer's instructions. Post‐staining, the tissues were decolorized in 75% ethanol to facilitate imaging. ROS accumulation was evaluated using NBT staining. Briefly, seeds were vacuum‐infiltrated (0.08 MPa, 15 min) with 0.2% (v/v) NBT working solution prepared from the BCIP/NBT Chromogen Kit (PR1100, Solarbio, Beijing, China) and incubated at 25°C for 8–24 h in darkness. Photographic imaging was performed using a *Nikon* SMZ800N stereo microscope equipped with a Nikon DS‐Fi3 camera. For starch grain observation, seeds from *Oscpn10a* mutants, *OsCPN10a* overexpression lines, and the wild‐type parent were transversely sectioned. The cross sections of the seed endosperm were examined using a Nikon SMZ800N stereo microscope equipped with a Nikon DS‐Fi3 camera and scanning electron microscope (Hitachi S‐3500N).

### Endogenous substance measurement

To measure endogenous substances, TNG67, *OsCPN10a* overexpression lines, and *Oscpn10a* mutant seeds subjected to artificial aging for durations of 0, 7, 14, or 21 d were used. The embryos were collected and subjected to measurement of apparent amylase, MDA, H_2_O_2_ content, and α‐amylase, SOD, POD, CAT, and APX activity. The endogenous substance contents were measured using absorbance methods with spectrophotometry, employing the corresponding assay kits from Beijing Solarbio Science Technology (Beijing, China).

### Phylogenetic analysis

The entire amino acid sequences of OsCPN10a were used as the query to search for homologous proteins in the NCBI website (https://blast.ncbi.nlm.nih.gov/Blast.cgi) and GBI website (https://phytozome.jgi.doe.gov). All chaperone proteins were clustered using ClustalX, and the phylogenetic tree was generated by MEGA7 based on the Neighbor‐Joining method and bootstrap analysis (1,000 replicates).

### Subcellular localization

For subcellular localization, the rice protoplasts were prepared and transfected with *OsCPN10a‐*nGFP fusion vectors co‐expressed with organelle markers, including endoplasmic reticulum (mCherry‐HDEL), peroxisome (mCherry‐SKL), and nucleus (mCherry‐D53) by the PEG method, then cultured at 28°C for 16 h (Zhang et al., 2011). The *OsCPN10a‐*nRFP, *OsCPN10b‐*nGFP, *OsHSP60‐3B‐*nGFP, *OsABIL1‐*nGFP, *OsPYL10‐*nGFP, *OsPYL10‐*nRFP, or *OsCPN20‐*nRFP fusion vectors were also co‐expressed in rice protoplasts. The GFP and RFP fluorescence was observed using the TCS SP8 STED 3X laser confocal microscope (Leica, Germany).

### UPLC measurement of ABA concentration

For ABA quantification, frozen embryos (≈ 200 mg fresh weight per replicate) at 0, 6, 12, 24, 36, and 72 h post‐imbibition were immediately snap‐frozen in liquid nitrogen and pulverized with 3 mm stainless‐steel beads in a Tissue Lyser (30 Hz, 2 min). Metabolites were extracted in 2 mL of the extraction solvent (methanol:ethyl acetate:acetic acid = 50:50:1) containing 20 ng/mL ABA (Solarbio, Cat. A8060) as internal standard and vortexed for 5 s. After sonication (4°C, 10 min) and overnight incubation (−20°C), samples were clarified (14,000 *g*, 15 min, 4°C) and the supernatant filtered with a 0.22 μm membrane filter. Three biological replicates were conducted. The ABA concentration was quantified using ultra‐high‐performance liquid chromatography on a Waters ACQUITY UPLC H‐Class system, equipped with a Waters C18 column (2.1 × 150 mm, 1.8 μm). The mobile phase comprised solvent A (water containing 0.1% formic acid and 5 mM ammonium acetate aqueous solution) and solvent B (acetonitrile containing 0.1% formic acid). The gradient elution program was as follows: from 0 to 2.0 min, solvent A phase remains at 90%; from 2.0 to 5.0 min, solvent A decreased from 90% to 20%; from 5.0 to 8.0 min, solvent A was maintained at 20%; and from 8.0 to 10.0 min, solvent A decreased from 20% to 90%. The flow rate was set at 0.3 mL/min, the column temperature was maintained at 35°C, the sample injection volume was 2.0 μL, and the detection wavelength was set at 265 nm.

### Seed germination analysis following ABA treatment

Fifty seeds each of the *Oscpn10a*, *Oscpn20*, *Oshsp60‐3b* mutants, the *OsCPN10a* overexpression lines, and the wild‐type parent TNG67 or ZH11 were surface‐sterilized and sown on sterile water or 1/2MS medium supplemented with 0, 5, 10, or 20 μM ABA for germination assessment. The experiment was conducted with three biological replicates. Seeds were incubated under a 14‐h light/10‐h dark cycle at 30°C and 80% relative humidity, with a light intensity of 100 μmol m^−2^ s^−1^. The number of germinated seeds was recorded daily, and the GP, GR, GI, root length, and shoot length were measured and calculated.

### Total protein extraction and western blot

The recombinant *OsCPN10a*‐GFP, *OsPYL10*‐GFP vector, and the control construct were subsequently transformed into rice protoplasts. Total protein was then extracted from the protoplasts. Protoplasts were harvested by centrifugation at 1,500 rpm for 3 min. Total protein was extracted with protein extraction buffer (50 mM Tris‐HCl at pH 7.5, 150 mM NaCl, 5 mM EDTA, 0.2% NP‐40, 0.1% Triton X‐100, and Complete protease inhibitor cocktail, Roche), usually 200–300 μL for 1 mL protoplasts (approximately 2 × 10^6^ cells). The extracts were then centrifuged at 16,000 rpm for 15 min at 4°C, and the supernatants were collected for WB analysis. The OsCPN10a‐GST fusion protein was expressed in *Escherichia coli* Rosetta (DE3) and purified with Anti‐GST beads (17527901, Cytiva, Shanghai, China). About 20 μg of total protein per sample was analyzed by SDS‐PAGE. WB analysis was performed with a monoclonal mouse anti‐GFP (66002‐1‐Ig, Proteintech, China, 1:5,000 dilution) or anti‐GST (M20007S, Abmart, China, 1:5,000 dilution) antibodies, along with an anti‐mouse secondary antibody (CWBIO, CW0102S, 1:10,000 dilution).

### Immunoprecipitation‐mass spectrometry (IP‐MS) assays

To identify proteins that interact with OsCPN10a in rice, we performed IP‐MS assays using OsCPN10a‐GFP isolated from transiently expressed in rice protoplasts (Jamge et al., 2018). Briefly, the OsCPN10a protein complex was immunoprecipitated with anti‐GFP beads (Sigma‐Aldrich, USA), and the immunoprecipitates were separated by SDS‐PAGE. Subsequently, the sample required a proteolytic step with trypsin before being injected into a mass spectrometer. Samples were analyzed using an online nano‐electrospray ionization LC–MS/MS system. The entire system consisted of a serially coupled EASY‐nano‐LC system (Thermo Scientific, MA, USA) and an Orbitrap Fusion Lumos Mass Spectrometer (Thermo Scientific). A 4 μL aliquot of the peptide sample was loaded onto a trap column (Thermo Scientific Acclaim PepMap C18, 100 μm × 2 cm) at a flow rate of 4 μL/min, followed by gradient separation on an analytical column (Acclaim PepMap C18, 75 μm × 15 cm) over 90 min. The column flow rate was controlled at 300 nL/min, and the electrospray voltage was set to 2 kV. Raw files were analyzed together using Maxquant (1.6.2.10). Proteins were identified using a target‐decoy approach by searching all MS/MS spectra against a concatenated forward/reversed version of the MSU‐Rice7.0_orypep7.0.pep.fasta database. Furthermore, MaxQuant incorporated relative, label‐free quantification (LFQ) for protein abundance assessment, resulting in a list of proteins present in the IP sample.

### Co‐IP assay

Co‐IP assays were performed as described previously (Luo et al., 2024). To perform the Co‐IP assay, *OsCPN10a*‐GFP and *OsCPN10a*‐Myc were transiently co‐expressed in rice protoplasts. Additionally, *OsPYL10*‐Myc was co‐transformed into rice protoplasts with *OsCPN10a‐*GFP, *OsHSP60‐3B*‐GFP, *OsCPN20*‐Myc, or *OsABIL1‐*GFP. Transformed protoplasts were harvested by centrifugation at 300 *g* for 5 min. Total protein was extracted using protein extraction buffer (25 mM Tris‐HCl, pH 7.5, 150 mM NaCl, 0.5% Triton X‐100, 1 mM EDTA, 1× PMSF (Lablead, China, P0754)) for 30 min on ice and then centrifuged at 15,000 *g* for 10 min at 4°C to remove aggregates. Protein samples were then incubated with Anti‐GFP beads (Lablead, China, GNA‐20‐400) at 4°C for 2 h with gentle agitation. Immunoblotting was performed using anti‐GFP (66002‐1‐Ig, Proteintech, China, 1:5,000 dilution), anti‐MYC (10828‐1‐AP, Proteintech, China, 1:5,000 dilution), and anti‐HA (51064‐2‐AP, Proteintech, China, 1:5,000 dilution), along with anti‐mouse secondary antibody (CWBIO, CW0102S, 1:10,000 dilution).

### Malate dehydrogenase activity assay

Chaperone activity was measured as the ability to prevent MDH denaturation under thermal‐denaturing conditions at 45°C. Malate dehydrogenase (MDH, EC 1.1.1.37, M007, Nanjing Jiancheng Bioengineering Institute, Nanjing, China) activity was measured spectrophotometrically at 340 nm and 45°C using the commercial MDH Assay Kit (A021‐2‐2, Nanjing Jiancheng Bioengineering Institute) following the manufacturer's instructions. Briefly, 20 μL of freshly extracted OsCPN10a‐GST protein supernatant was added to 200 μL of pre‐warmed reaction mixture containing NADH and oxaloacetate. MDH (0.3 μM) was incubated in the absence or presence of purified recombinant OsCPN10a‐GST protein, in which various molar ratios of MDH to OsCPN10a‐GST (1:1, 1:3, or 1:5) were examined. The oxidation of NADH was monitored for 3 min (ε = 6.22 mM^−1^ cm^−1^) and activity expressed as nM NADH oxidized min^−1^ mg^−1^ protein. BSA was used as a negative control.

### Yeast two‐hybrid assay

The yeast two‐hybrid assays were conducted according to the protocol specified in the Matchmaker Gold Yeast Two‐Hybrid System User Manual (Clontech, Mountain View, CA, USA). The full‐length coding sequences of *OsCPN10a* and *OsPYL10* were cloned into pGBKT7 (Clontech, Dalian, China) as a “bait” vector, and the full‐length coding sequences of *OsCPN10b*, *OsCPN20*, *OsHSP60‐3B*, and *OsABIL1* were inserted into pGADT7 (Clontech) as a “prey” vector. The two plasmid vectors were co‐transformed into the yeast strain Y2HGold (Weidi Biotechnology, Shanghai, China). Transformants were spotted on synthetic defined (SD) medium lacking Ura and Leu (Coolaber, Beijing, China). After 28°C cultivation for 3 d, five colonies were randomly selected, spotted on SD/−Leu−Trp−His−Ade media (Coolaber), and incubated for 3 d. The primers used for cloning are listed in Table S1.

### Bimolecular fluorescence complementation (BiFC) assay

For subcellular localization in rice protoplasts, the protoplasts were prepared and transfected with *OsCPN10a‐*nYFP, *OsCPN10a‐*cYFP, *OsHSP60‐3B‐*cYFP, *OsABIL1‐*cYFP, *OsPYL10‐*cYFP, *OsPYL10‐*nYFP, *OsCPN20‐*cYFP, and *OsCPN20‐*nYFP fusion vectors by the PEG method, then cultured at 28°C overnight (Zhang et al., 2011). The YFP fluorescence was observed using the TCS SP8 STED 3X laser confocal microscope (Leica, Germany). The experiments have three biological replicates with similar results.

### Luciferase complementation imaging assays

Full‐length CDSs of *OsCPN10a*, *OsCPN10b*, *OsCPN20*, and *OsHSP60‐3B* were cloned into the *NlucN‐HA* and *NlucC‐FLAG* vectors driven by the 35S promoter. All the construct combinations were transiently transformed into rice protoplasts with three independent replicates as described in a previous report (Zhang et al., 2011). Both the firefly luciferase (LUC) and REN activity were measured using a DLR assay kit (Promega, Madison, WI, USA) on a Centro XS3 LB 960 High Sensitivity Microplate Luminometer (Berthold Technologies, Bad Wildbad, Germany) and imaged with Nightshade LB985 Plant *in vivo* imaging system (Berthold Technologies, Bad Wildbad, Germany). To calculate the relative LUC activity (LUC/REN), the LUC activity was normalized to that of REN. Full‐length CDS of *OsCPN10a* and *OsPYL10* were cloned into the *pCAMBIA1300‐35SCLUC* vector. The full‐length CDS of *OsCPN10b*, *OsCPN20*, *OsHSP60‐3B*, and *OsABIL1* were inserted into the *pCAMBIA1300‐35SNLUC* vector. All constructs were transformed into *A. tumefaciens* strain GV3101 (*pSoup‐p19*) and co‐infiltrated into 5‐ to 6‐week‐old *N. benthamiana* leaves as previously described (Fang et al., 2025). The treated leaves were sprayed with 1 mM luciferin (GoldBio, St Louis, MO, USA), incubated in darkness for 5 min, and imaged with Vilber Newton 7.0 Biochemiluminescence imaging system (Vilber Lourmat, France) at 72 h after infiltration. Luciferin (1 mM) was sprayed onto the leaves, and the plants were kept in the dark for 2–5 min. The experiments have three biological replicates with similar results. LUC images were captured by a Vilber Newton 7.0 Bio with a cooled CCD imaging apparatus (VILBER LOURMAT, Marne‐la‐Vallée, France). Photon flux was quantified with ViNeo software v2.0.

### Statistical analysis

All assays were conducted with at least three replicates per line. GraphPad Prism 9 software was used to calculate the means ± standard deviation (*SD*) from three independent experiments. Statistical significance was determined by a two‐sided Student's *t*‐test, with significance levels defined as follows: *P < 0.05, **P < 0.01, ***P < 0.001, and ****P < 0.0001. Different letters on the bars indicate significant differences by one‐way analysis of variance (ANOVA) analysis–Duncan test. The band intensity of immunoblotting was measured with ImageJ. The primers used for transgenic assay, RT‐qPCR assay, subcellular localization, LUC, BiFC, Co‐IP assay, and yeast transformation are listed in Table S1.

### Generative AI usage statement

During the preparation of this work, the authors utilized Kimi (https://www.kimi.com/), a generative AI tool, for the purpose of translation between Chinese and English and manuscript language polishing. After using this tool/service, we carefully reviewed and revised the content where necessary and took full responsibility for the final version of the publication.

## CONFLICTS OF INTEREST

The authors declare no conflicts of interest.

## AUTHOR CONTRIBUTIONS

S.F.L. performed most of the research and drafted the manuscript. Y.D.W. and J.L.W. generated the *OsCPN10a* knockout mutants; Y.D.W. also provided the proteomics dataset and technical assistance. S.F.L. and J.L.W. conducted the *OsCPN10a* overexpression experiments. Y.S.Z. managed the field cultivation of transgenic seeds. T.C. and K.Y.L. performed the LUC assays, some BIFC, and Co‐IP experiments. S.Z. and P.B.Y. performed some Co‐IP experiments and participated in manuscript revision. F.X.W., H.G.X., L.P.C., and Q.H.C. provided technical assistance. H.A.X. supervised the project. J.F.Z. designed the experiments, supervised the study, and revised the manuscript. All authors have read and approved the contents of this paper.

## Supporting information

Additional Supporting Information may be found online in the supporting information tab for this article: http://onlinelibrary.wiley.com/doi/10.1111/jipb.70230/suppinfo

**Figure S1.** OsCPN10a is a key regulator of seed aging tolerance in rice
**Figure S2.** Genotyping of *Oscpn10a* mutants and phenotyping of wild‐type control (TNG67), *Oscpn10a* mutants, and *OsCPN10a* overexpression lines (*OE‐1* and *OE‐2*)
**Figure S3.** Knockout of *OsCPN10a* delayed germination following dormancy‐breaking treatment
**Figure S4.** *OsCPN10a* positively regulates seed aging and heat resistance
**Figure S5.** Phylogenetic analysis of HSP10 and CPN10 in different species
**Figure S6.** Analysis of sequence homology and conserved domains of OsCPN10a
**Figure S7.** Identification of the candidate pathway for *OsCPN10a* regulates seed germination
**Figure S8.** Western blot analysis of OsCPN10a‐GFP expressed in rice protoplasts in IP‐MS and prokaryotically expressed OsCPN10a‐GST
**Figure S9.** Partial candidate interactors identified from the OsCPN10a IP‐MS screen
**Figure S10.** Relative expression of *OsCPN10b*, *OsCPN20*, and *OsHSP60‐3B* in the *Oscpn10a* mutants and their co‐localization with OsCPN10a
**Figure S11.** OsCPN10a promoted the stability of OsCPN20 and OsHSP60‐3B *in vivo*
**Figure S12.** Genotyping of *Oscpn20* mutants in the ZH11 background
**Figure S13.** Genotyping of *Oshsp60‐3b* mutants in the ZH11 background
**Table S1.** Sequences of all primers used in this study

## Data Availability

Gene sequence information can be found in the RAP‐DB database (http://rice.plantbiology.msu.edu/), following accessions *OsCPN10a* (LOC_Os 03g25050), *OsZFP15* (LOC_Os03g60570), *OsZFP182* (LOC_Os03g60560), *OsNCED3* (LOC_Os03g44380), *OsNCED4* (LOC_Os07g05940), *OsNCED5* (LOC_Os12g42280), *OsABA8ox1* (LOC_Os02g47470), *OsABA8ox2* (LOC_Os08g36860), *OsABA8ox3* (LOC_Os09g28390), *OsABI3* (LOC_Os01g68370), *OsABI4* (LOC_Os05g28350), *OsABI5* (LOC_Os01g64000), *OsPYL4* (LOC_Os03g18600), *OsPYL10* (LOC_Os10g42280), *OsCPN10b* (LOC_Os07g44740), *OsCPN20* (LOC_Os06g09679), *OsHSP60‐3B* (LOC_Os10g32550), *OsPP2C53* (LOC_Os05g51510), *OsPP2C30* (LOC_Os03g16170), and *OsABIL1* (LOC_Os01g64730). The raw data that support the findings of this study are available from the corresponding author upon reasonable request.
