# Supplementary material for: OsCPN10a, cooperating with OsCPN20 and OsHSP60‐3B negatively regulate ABA signaling and enhance seed storability in rice
Source: J Integr Plant Biol. 2026 Mar 16;68(7):2242–63. doi: 10.1111/jipb.70230 (PMC13326984; doi:10.1111/jipb.70230)
Supplement: Supplementary file 1 — Figure S1. OsCPN10a is a key regulator of seed aging tolerance in rice Figure S2. Genotyping of Oscpn10a mutants and phenotyping of wild‐type control (TNG67), Oscpn10a mutants, and OsCPN10a overexpression lines (OE‐1 and OE‐2) Figure S3. Knockout of OsCPN10a delayed germination following dormancy‐breaking treatment Figure S4. OsCPN10a positively regulates seed aging and heat resistance Figure S5. Phylogenetic analysis of HSP10 and CPN10 in different species Figure S6. Analysis of sequence homology and conserved domains of OsCPN10a Figure S7. Identification of the candidate pathway for OsCPN10a regulates seed germination Figure S8. Western blot analysis of OsCPN10a‐GFP expressed in rice protoplasts in IP‐MS and prokaryotically expressed OsCPN10a‐GST Figure S9. Partial candidate interactors identified from the OsCPN10a IP‐MS screen Figure S10. Relative expression of OsCPN10b, OsCPN20, and OsHSP60‐3B in the Oscpn10a mutants and their co‐localization with OsCPN10a Figure S11. OsCPN10a promoted the stability of OsCPN20 and OsHSP60‐3B in vivo Figure S12. Genotyping of Oscpn20 mutants in the ZH11 background Figure S13. Genotyping of Oshsp60‐3b mutants in the ZH11 background Table S1. Sequences of all primers used in this study [file JIPB-68-2242-s001.doc]

# Supplemental information

**OsCPN10a, cooperating with OsCPN20 and OsHSP60-3B negatively regulate ABA signaling and enhance seed storability**

Sufeng Liao^1,2,3#^, Yidong Wei^1,3#^, Yongsheng Zhu^1,3^, Kunyang Li^1,3^, Ting Chen^1,2,3^, Shuai zhao^4^, Fangxi Wu^1,2,3^, Jinlan Wang^2,3^, Pengbo Yu^2^, Hongguang Xie^1,2,3^, Liping Chen^2,3^, Qiuhua Cai^2,3^, Huaan Xie^1,2,3^ and Jianfu Zhang^1,2,3^,*

^1^Rice Research Institute, Fujian Academy of Agricultural Sciences, Fuzhou 350019, China

^2^Cross-straits Agricultural Technology Cooperation Center, College of Agriculture, Fujian Agriculture and Forestry University, Fuzhou 350002, China

^3^State Key Laboratory of Ecological Pest Control for Fujian and Taiwan Crops/Key Laboratory of Germplasm Innovation and Molecular Breeding of Hybrid Rice for South China, Ministry of Agriculture and Affairs, P.R. China/Incubator of National Key Laboratory of Germplasm Innovation and Molecular Breeding between Fujian and Ministry of Sciences and Technology/Fuzhou Branch, National Rice Improvement Center of China/Fujian Engineering Laboratory of Crop Molecular Breeding/Fujian Key Laboratory of Rice Molecular Breeding, Fuzhou 350003, China

^4^Key Laboratory for Basic and Applied Research of Bayu Prescriptions and Herbs, College of Chinese Materia Medica, Chongqing University of Chinese Medicine，Chongqing 361000, China

^#^ These authors contributed equally to this article.

^*^Correspondence: Jianfu Zhang ([jianfzhang@163.com](mailto:jianfzhang@163.com))


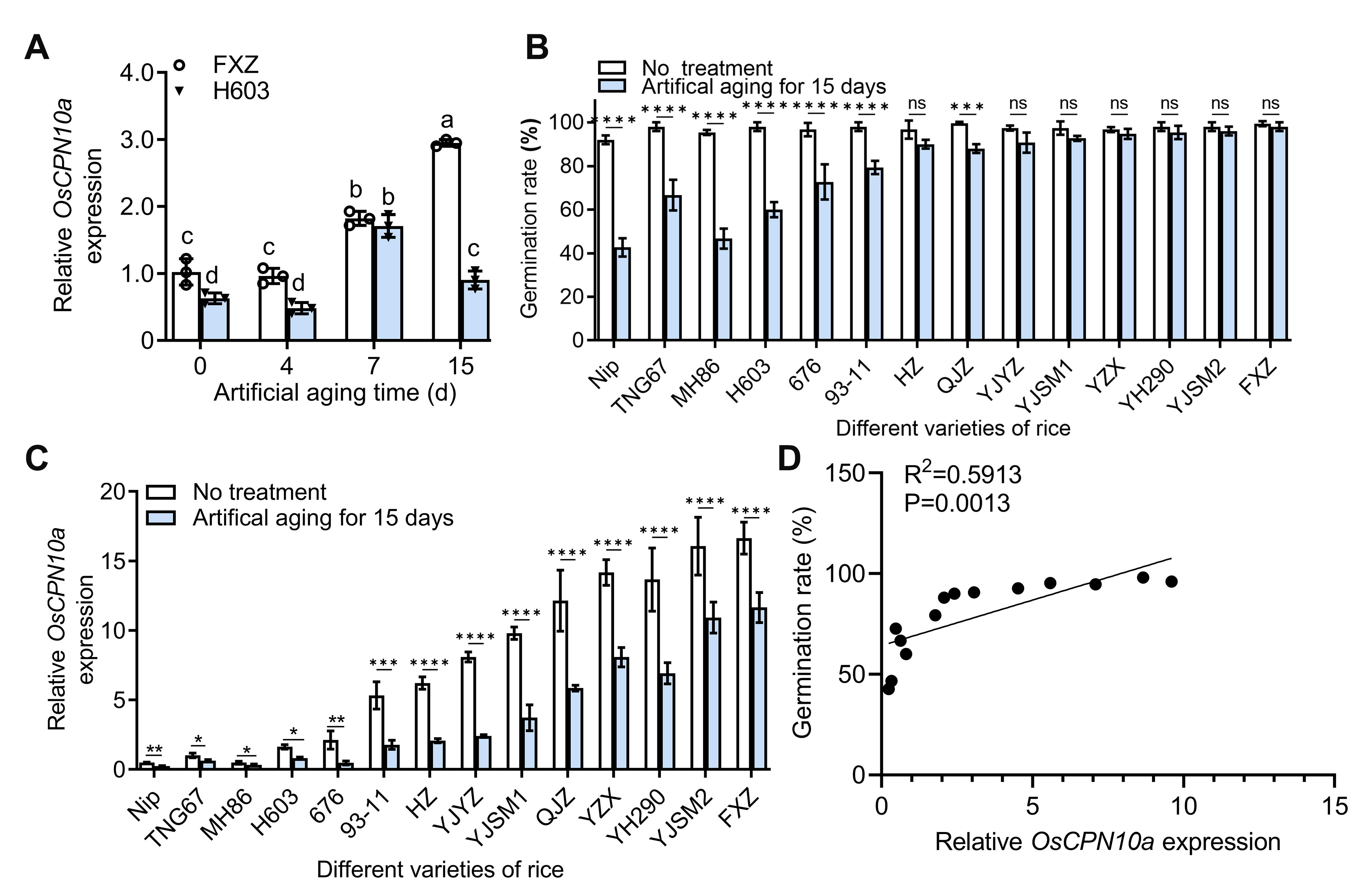


**Figure S1.** ***OsCPN10a* as a key regulator of seed aging tolerance in rice**

**(A)** Relative expression of *OsCPN10a* in FXZ and H603 embryos during artificial aging for 0, 4, 7, or 15 days. Data represent mean ± standard deviation (*SD*) from three independent experiments (biological replicates), with three technical replicates per experiment. Different letters denote significant difference at *P* < 0.05 by two-way ANOVA. **(B)** Germination rate of different rice cultivars before and after artificial aging for 15 days. **(C)** Relative expression of *OsCPN10a* in different rice cultivars embryos treated with or without artificial aging for 15 days. *OsACTIN150* was used as an internal control. In B, C, data were presented as means ± *SD* (*n* = 3; **P* < 0.05; ***P* < 0.01; ****P* < 0.001; ****P* < 0.0001, *****P* < 0.0001; Student’s t-test). **(D)** Correlation analysis between germination rates of seeds from 14 rice cultivars after high-temperature storage and their corresponding *OsCPN10a* transcript levels. *n* = 14 cultivars. Pearson correlation coefficient (r) and two-tailed *P*-value were calculated to assess the significance of the linear relationship. Nip: Nipponbare, TNG67: Tainong67, Minghui 86: MH86, Qingjingzhan: QJZ, Huazhan: HZ, Yujingyouzhan: YJYZ, Yujingshimiao: YJSM1, Yuzhenxiang: YZX, Yunhui 290: YH290, Yuejingshimiao: YJSM2, Fuxiangzhan: FXZ.


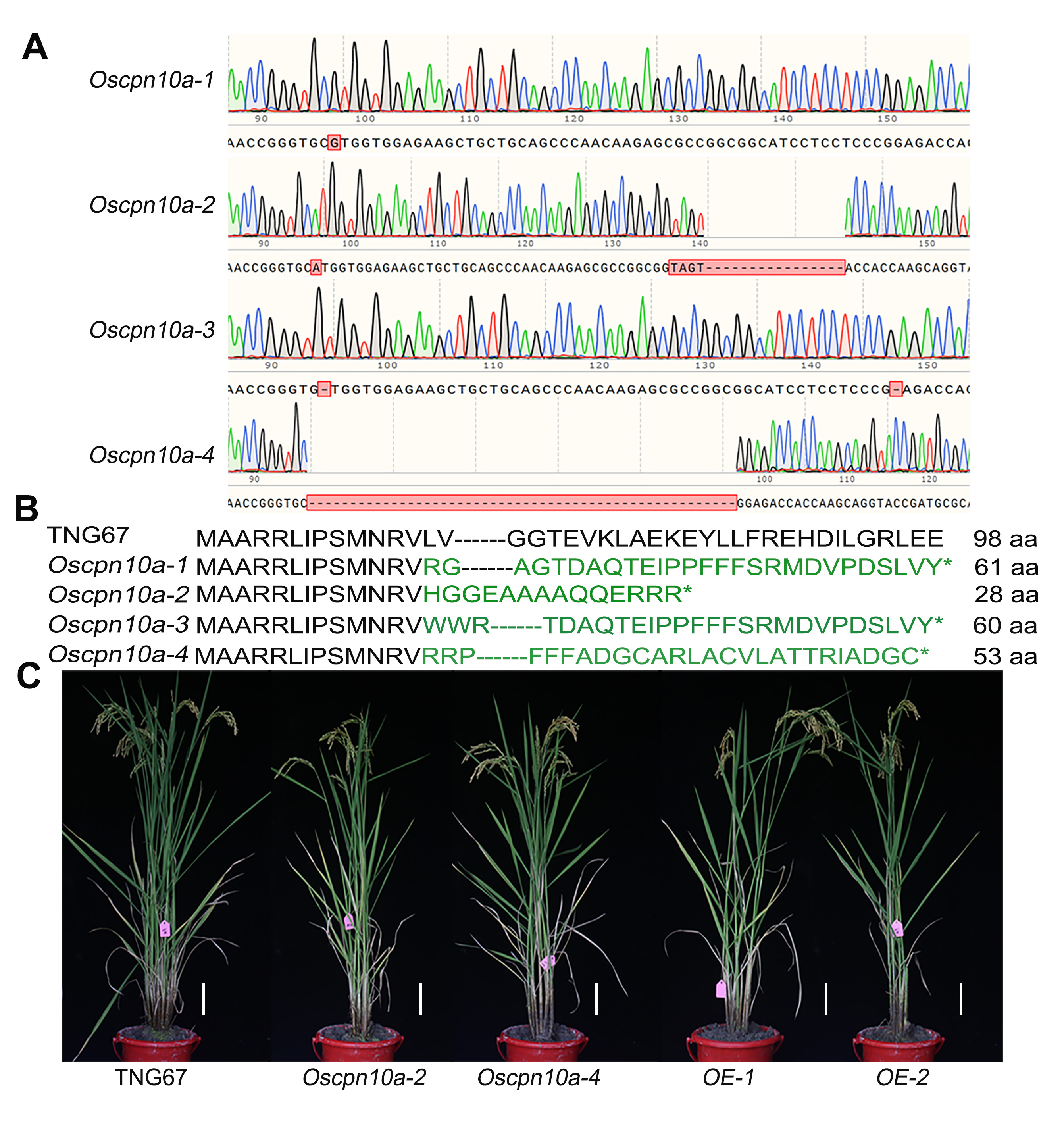


**Figure S2.** **Genotyping of *Oscpn10a* mutants and phenotyping of wild-type control (TNG67), *Oscpn10a* mutants, and *OsCPN10a* overexpression lines (*OE-1* and *OE-2*)**

**(A)** Comparison of sanger sequencing chromatograms between WT and *Oscpn10a* mutants.​ **(B)** Protein sequence of *OsCPN10a* in TNG67 and *Oscpn10a* mutants. **(C)** Phenotyping of TNG67 and *OsCPN10a* transgenic lines.​​ Scale bar, 10 cm.


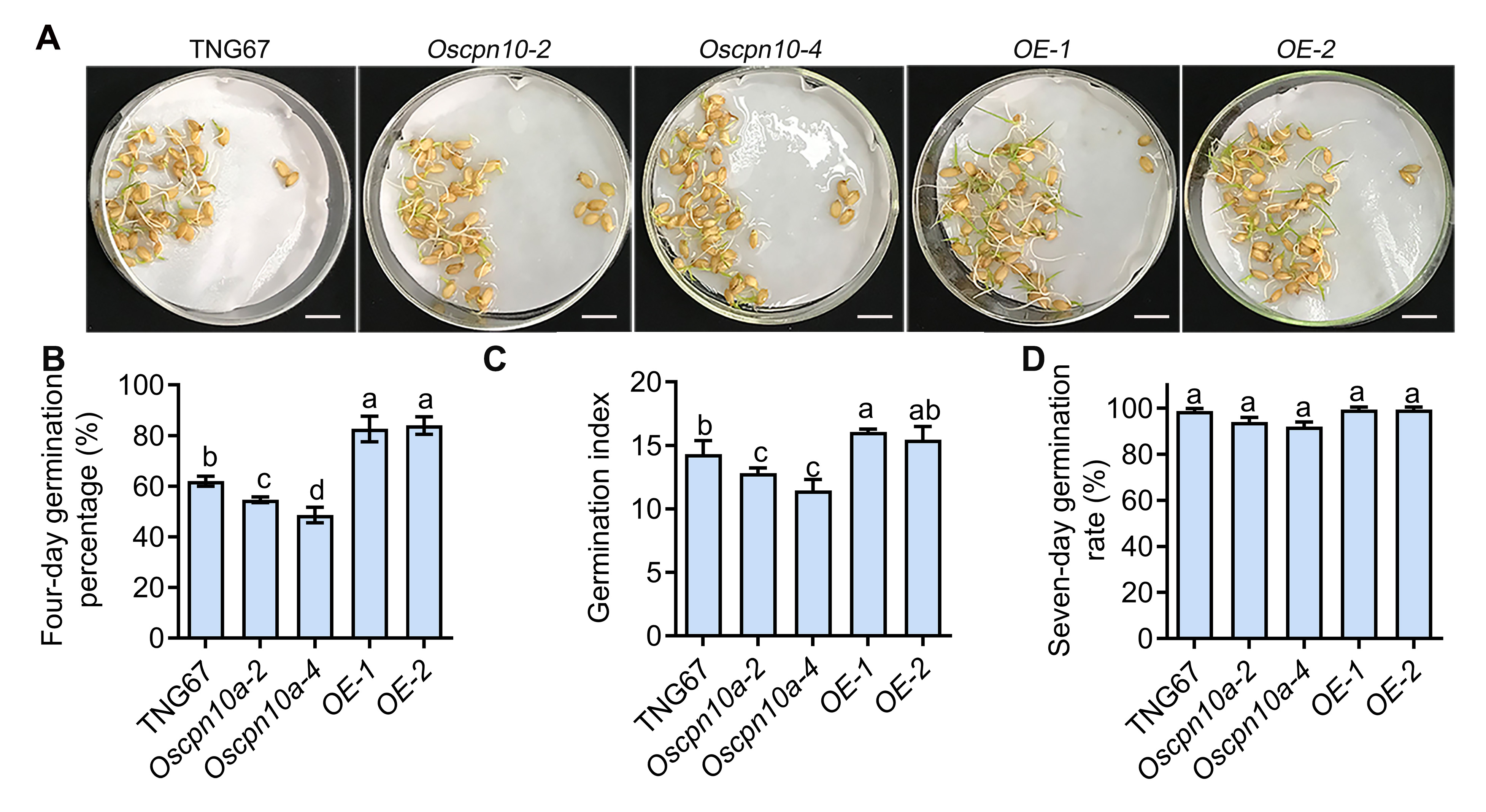


**Figure S3. Knockout of *OsCPN10a* delayed germination following dormancy-breaking treatment**

**(A)** Seed germination photographs of TNG67, the *Oscpn10a* mutants, and *OsCPN10a* overexpression lines for 7 days following 48 h of chemical dormancy, released with 0.5 mM GA₃ at 30℃. Scale bar, 1 cm. **(B-D)** Comparison of four-day germination percentage (GP), seven-day germination rate (GR), and germination index (GI) of wild-type TNG67, *Oscpn10a* mutants, and *OsCPN10a* overexpression lines after breaking dormancy. Data are given as means ± *SD* of three biological replicates (50 seeds per biological replicate), with three technical replicates per experiment. Different lowercase letters above the bars indicate significant differences (*P <* 0.05) based on two-way ANOVA.


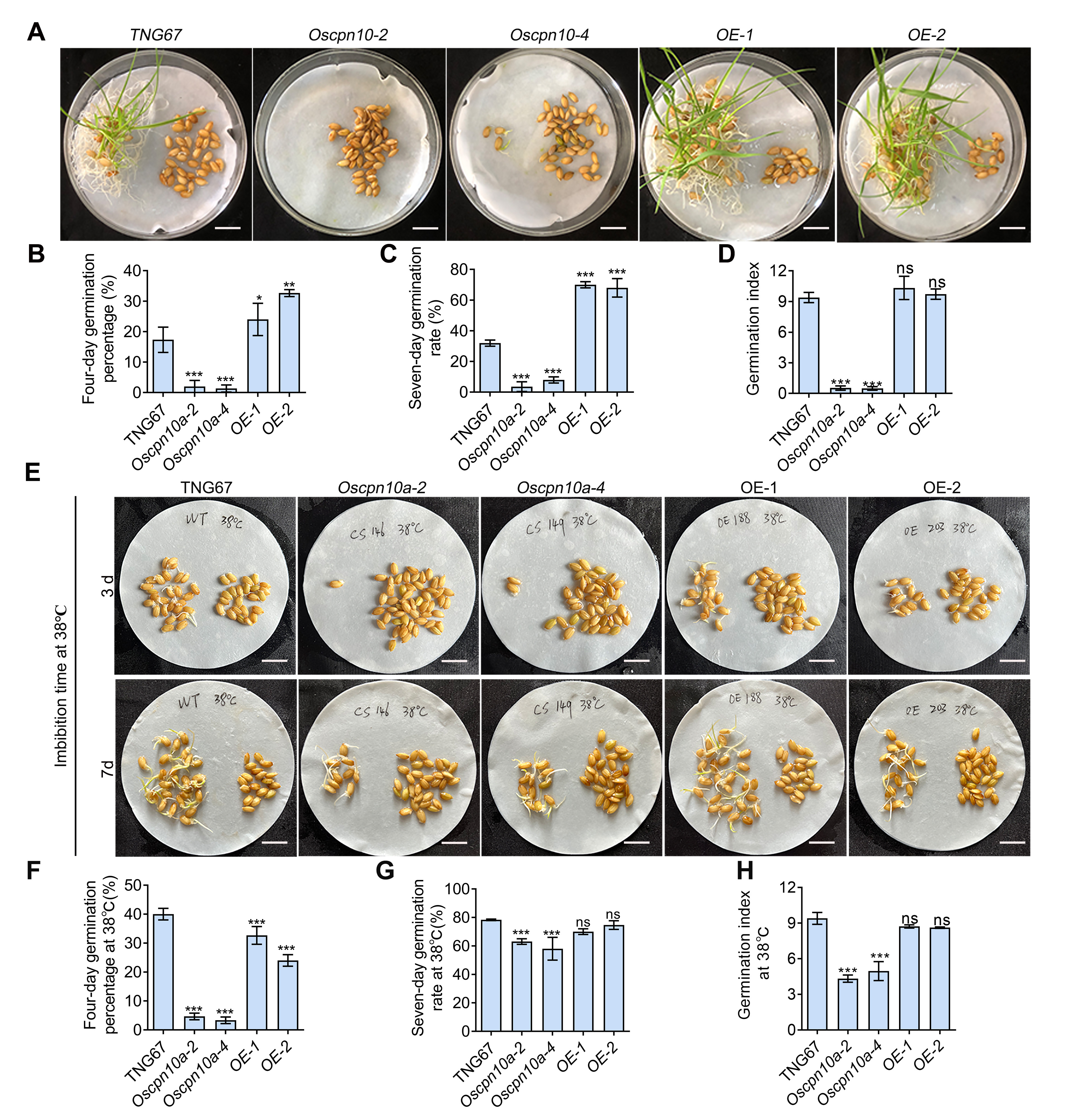


**Figure S4. *OsCPN10a* positively regulates seed aging and heat resistance**

**(A-D)** Assessment of aging resistance of TNG67, *Oscpn10a* mutants (*Oscpn10a-2* and *Oscpn10a-4*), and *OsCPN10a* overexpression lines. Photographs of seed germination of TNG67, *Oscpn10a* mutants and *OsCPN10a* overexpression lines for 7 days following natu­ral aging for 6 months (A). Scale bar, 1 cm. Comparison of GP, GR and GI of TNG67, *Oscpn10a* mutants and *OsCPN10a* overexpression lines after 7 days following natural aging for 6 months (B-D). **(E-H)** Assessment of heat resistance of TNG67, *Oscpn10a* mutants, and *OsCPN10a* overexpression lines. Seed germination photographs of TNG67, *Oscpn10a* mutants, and *OsCPN10a* overexpression lines under 38℃ (E). Scale bar, 1 cm. Comparison of seed GP, GR, and GI of WT, *Oscpn10a* mutants, and *OsCPN10a* overexpression lines (F-H). In B**–**D and F**–**H, the data are given as means ± *SD* of three biological replicates (50 seeds per biological replicate), with three technical replicates per experiment. **P* < 0.05, ***P* < 0.01, and ****P* < 0.01; Student’s *t*-test.


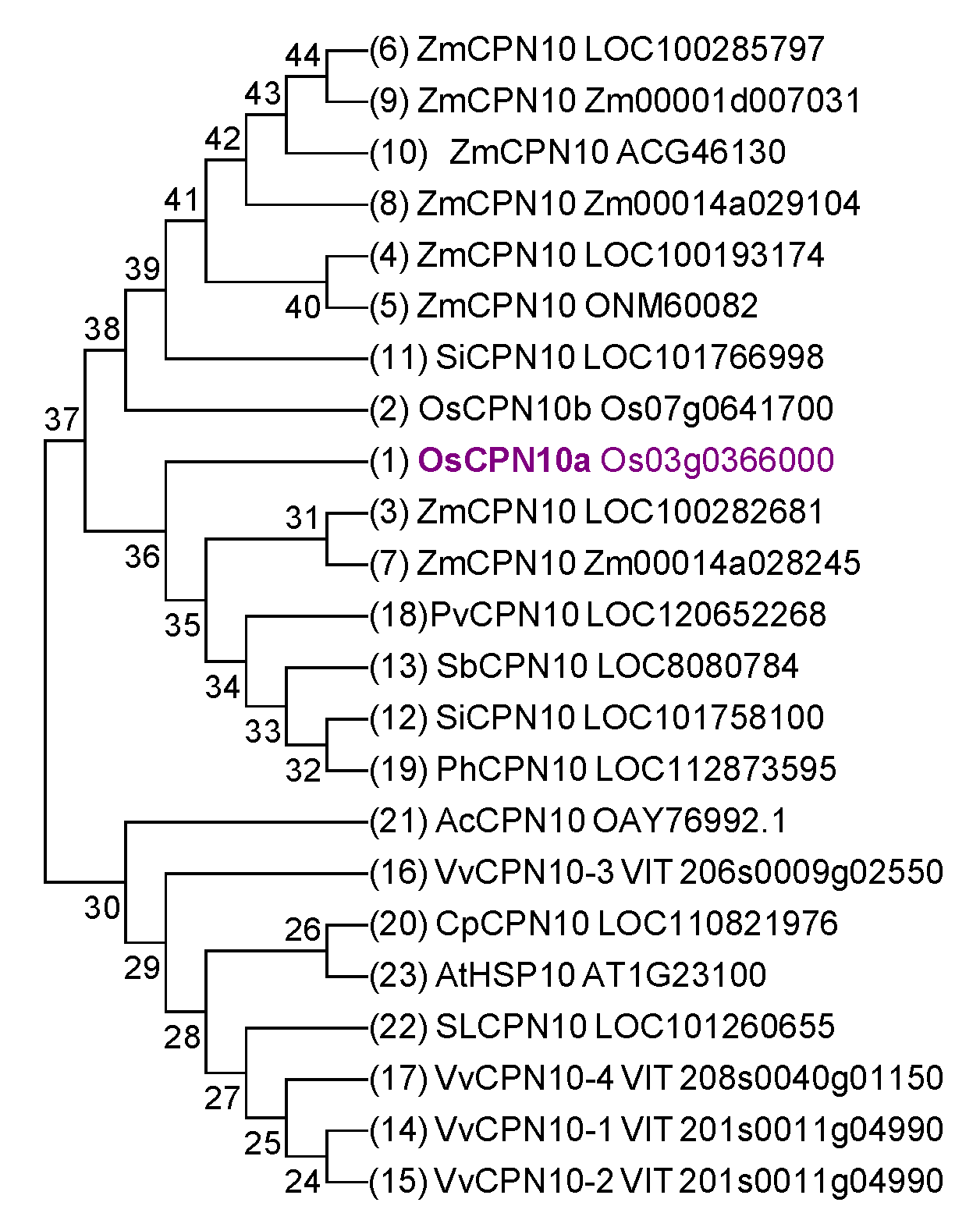


**Figure S5. Phylogenetic analysis of HSP10 and CPN10 in different species**


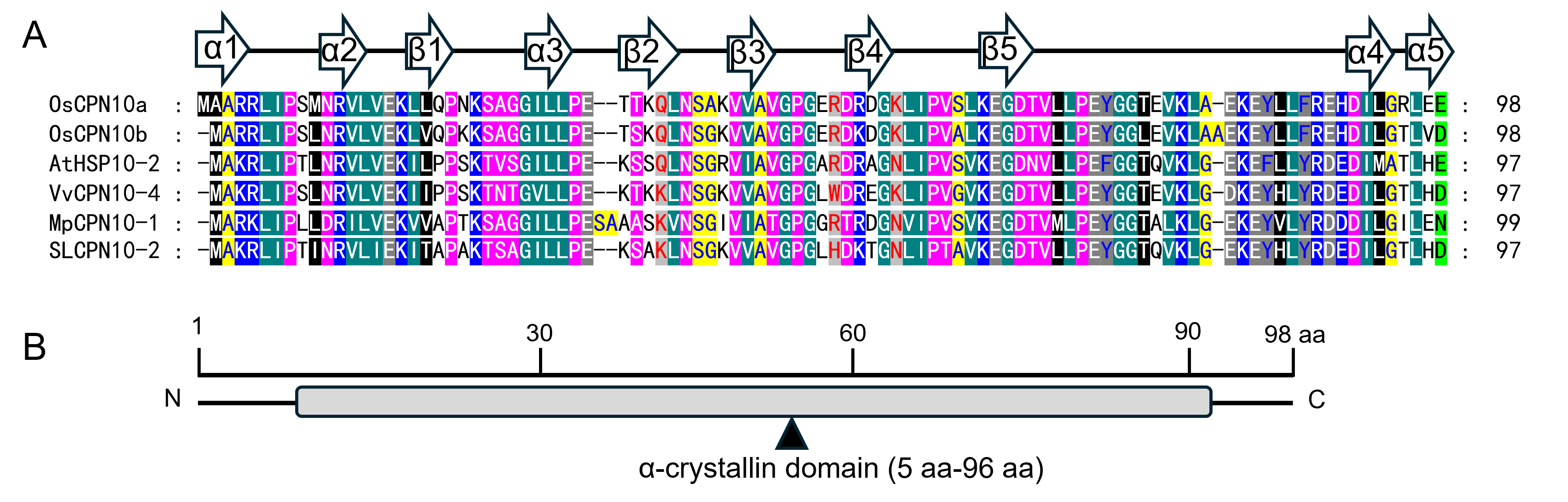


**Figure S6.** **Analysis of sequence homology and conserved domains of OsCPN10a**

**(A)** Analysis of OsCPN10a amino acid sequence homology. **(B)** *α*-crystallin domain of OsCPN10a. The accession numbers are as follows: OsCPN10b (Os07g0641700, *Oryza sativa*), AtHSP10 (AT1G23100, *Arabidopsis thaliana*), VvCPN10 (VIT_208s0040g01150, *Vitis vinifera*), MpCPN10 (Mapoly0005s0017, *Marchantia polymorpha*), SLCPN10 (KAL3338371, *Solanum stoloniferum***)**.


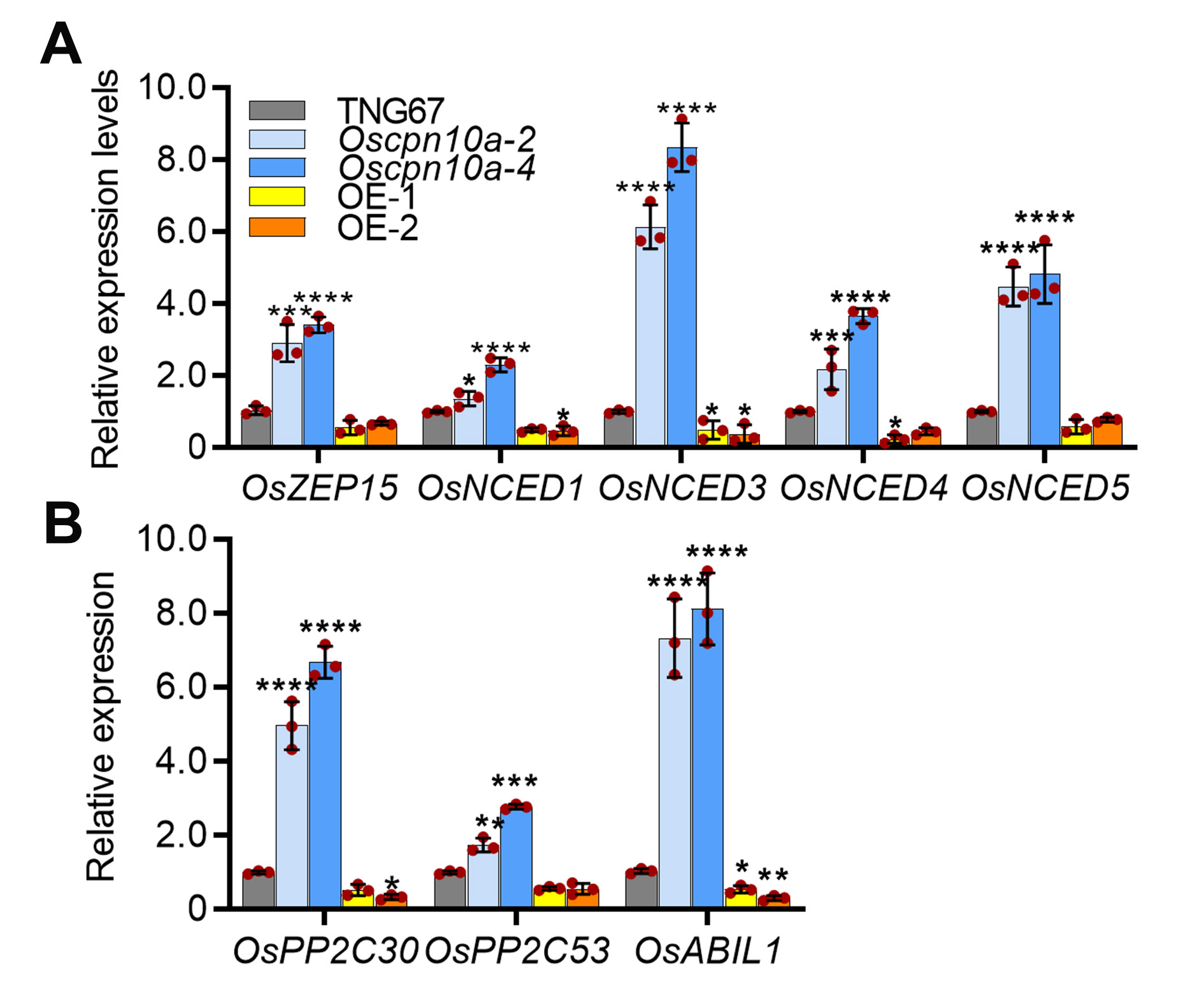


**Figure S7.** **Identification of the candidate pathway for OsCPN10a regulates seed germination**

**(A, B)** Relative expression of ABA degradation genes (*OsABA8ox1/2/3*) and ABA signaling genes (*OsPP2C30/53* and *OsABIL1*) in embryos of TNG67, *Oscpn10a* mutants and *OsCPN10a* overexpression lines. Relative expressions were represented by the fold-change relative to TNG67 expression. The gene expression was normalized to that of the *OsActin150* gene control. Expression at stage 1 was set to 1, and the relative expression levels were calculated for several genes. Data are presented as means ± *SD* from three biologically independent replicates (*n* = 3; **P* < 0.05,***P* < 0.01, and ****P* < 0.001; Student’s *t*-test).


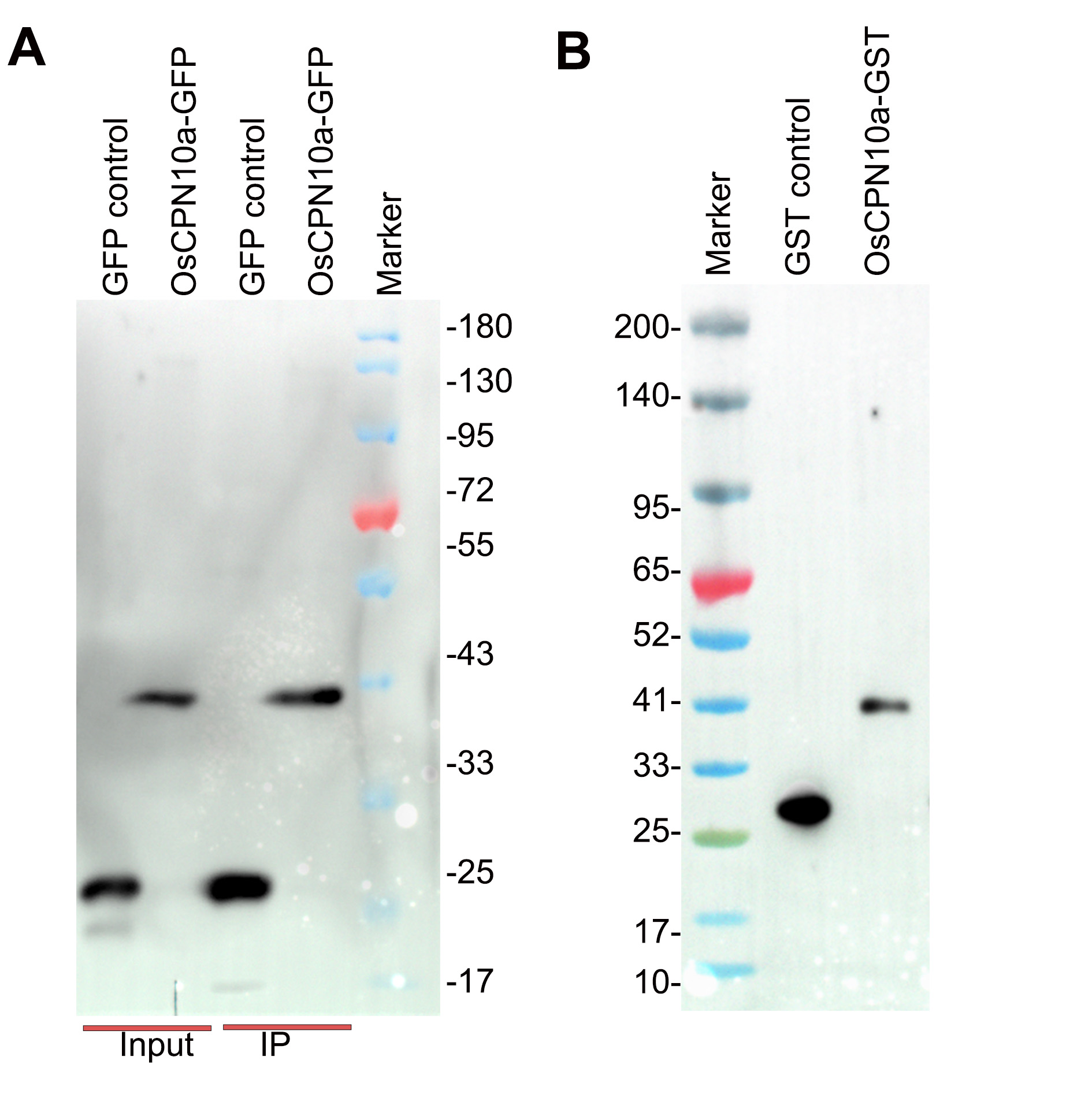


**Figure S8. Western blot analysis OsCPN10a-GFP expressed in rice protoplasts in IP-MS and prokaryotically expressed OsCPN10a-GST**

Western blot analysis OsCPN10a-GFP expressed in rice protoplasts in IP-MS (A) and prokaryotically expressed OsCPN10a-GST(B).


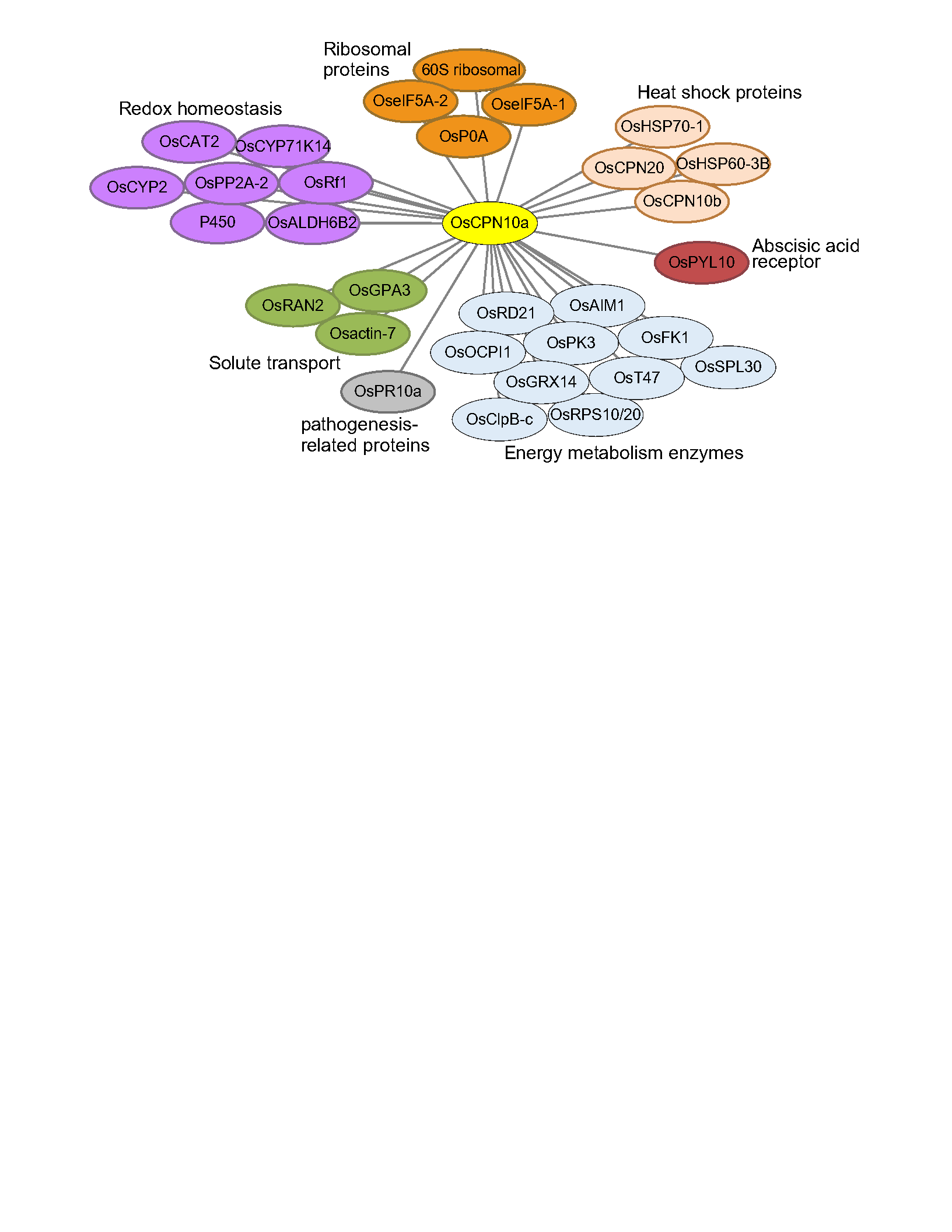


**Figure S9. Partial candidate interactors identified from the OsCPN10a IP-MS screen**

Interactome of the mediator complex network was constructed with the Cytoscape software.


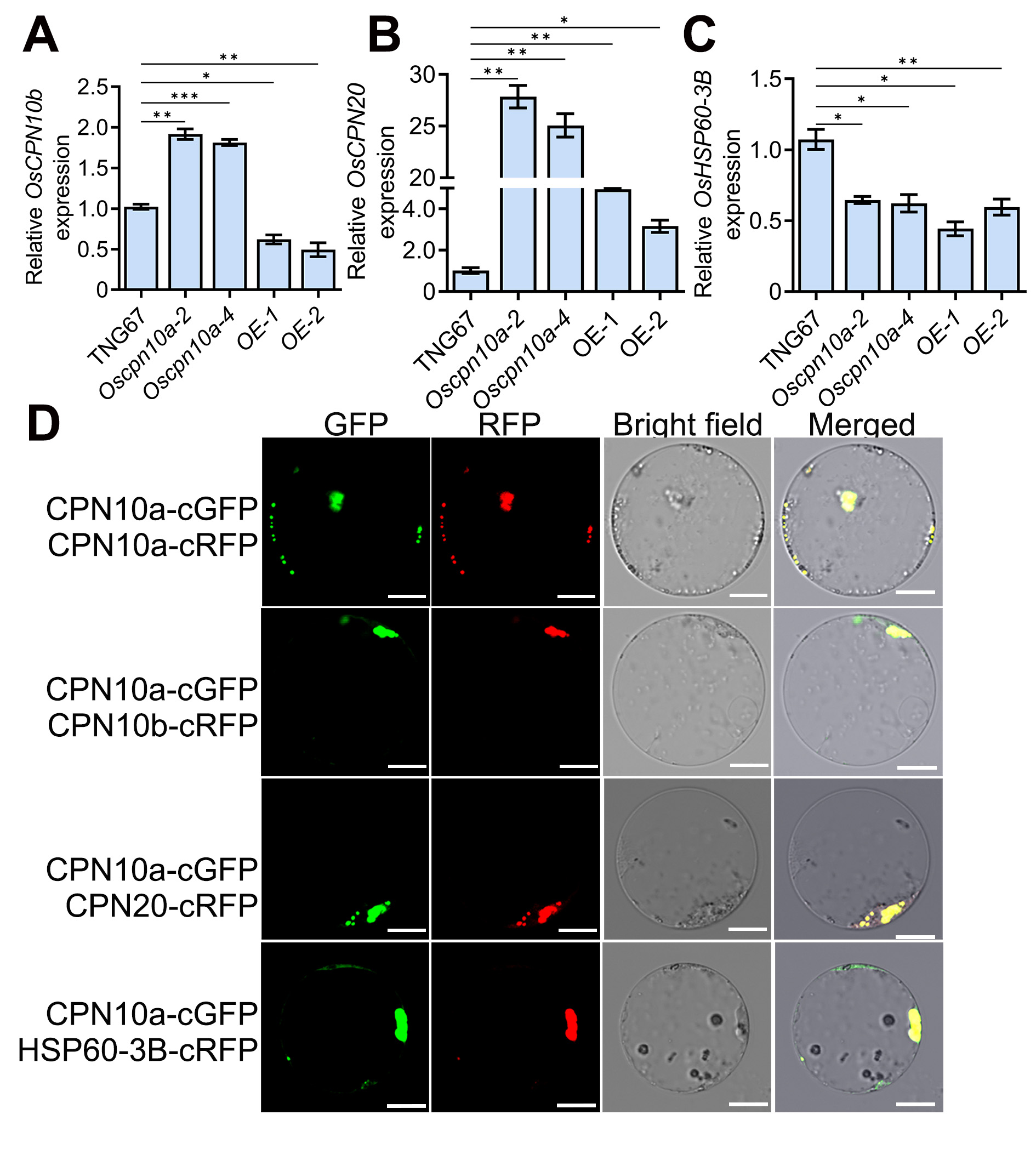


**Figure S10.** **Relative expression of *OsCPN10b*, *OsCPN20*, and *OsHSP60-3B* in the *Oscpn10a* mutants and their co-localization with OsCPN10a**

**(A-C)** Relative expression of *OsCPN10b*, *OsCPN20* and *OsHSP60-3B* in seed embryos of TNG67, *Oscpn10a* mutants and *OsCPN10a* overexpression lines. The relative expression levels were monitored by reverse transcription quantitative real-time PCR (RT-qPCR) against *Osactin150* gene control. Data represents means ± *SD* from three biologically independent replicates (*n* = 3; **P* < 0.05, ***P* < 0.01, ****P* < 0.001 and ns indicate *P* > 0.05; Student’s t-test). D) Subcellular co-localization of OsCPN10a-GFP with OsCPN10b-RFP, OsCPN20-RFP and OsHSP60-3B-RFP. Scale bar, 10 μm.


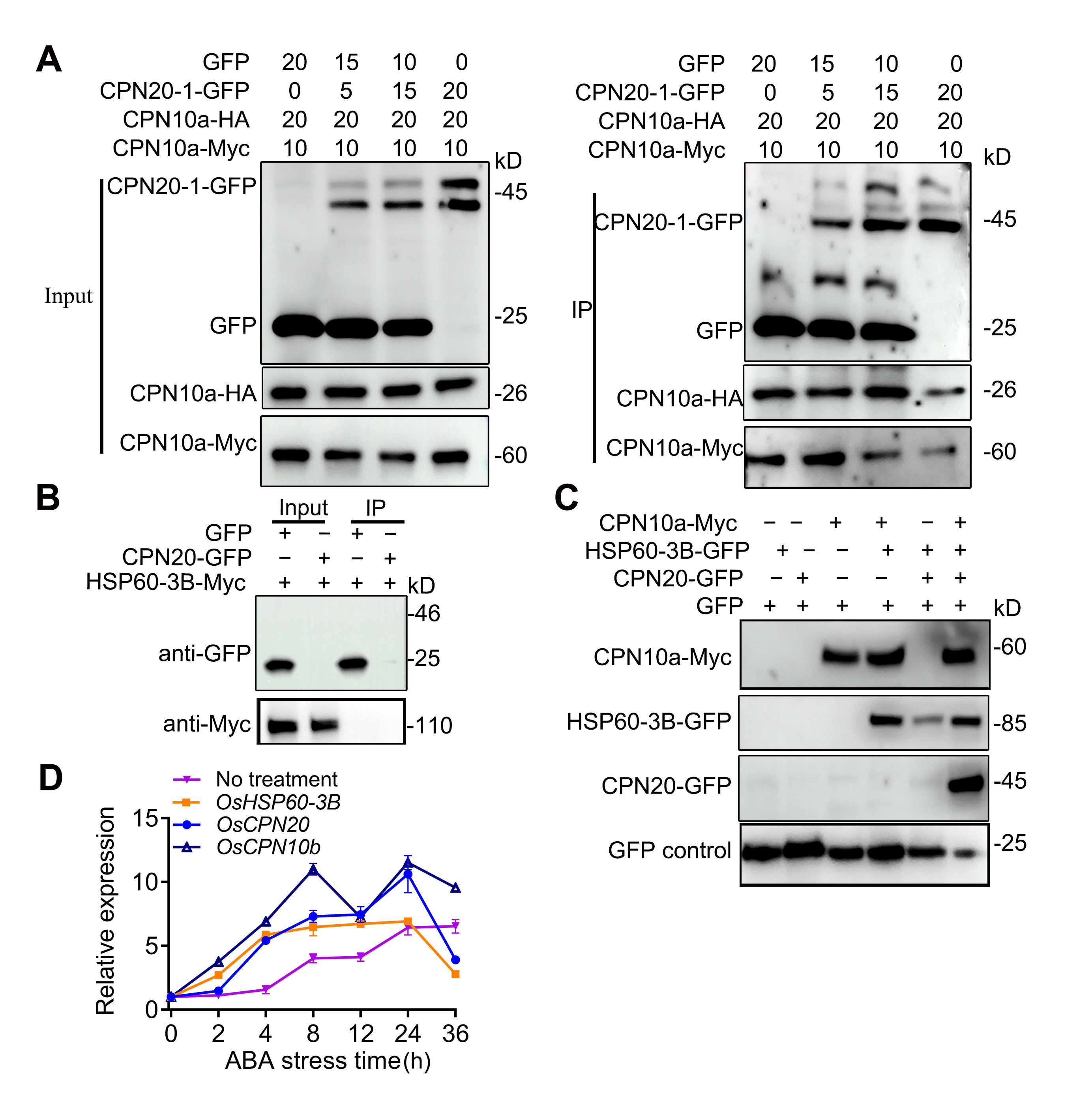


**Figure S11. OsCPN10a promoted the stability of OsCPN20 and OsHSP60-3B *in vivo***

**(A)** co-IP test verifies OsCPN20 inhibits the formation of OsCPN10a homodimers. **(B)** co-IP assays showed that OsCPN20 is unstable when co-expressed with OsHSP60-3B. **(C)** WB assays in rice protoplasts transiently expressed OsCPN10a-Myc, OsCPN20-GFP, and OsHSP60-3B-GFP fusion proteins.

**(D)** Relative expression of *OsCPN10b*, *OsCPN20* and *OsHSP60-3B* in TNG67 embryos at different time points during seed imbibition stages with ABA treatment. Data represents means ± *SD* from three biologically independent replicates (*n* = 3).


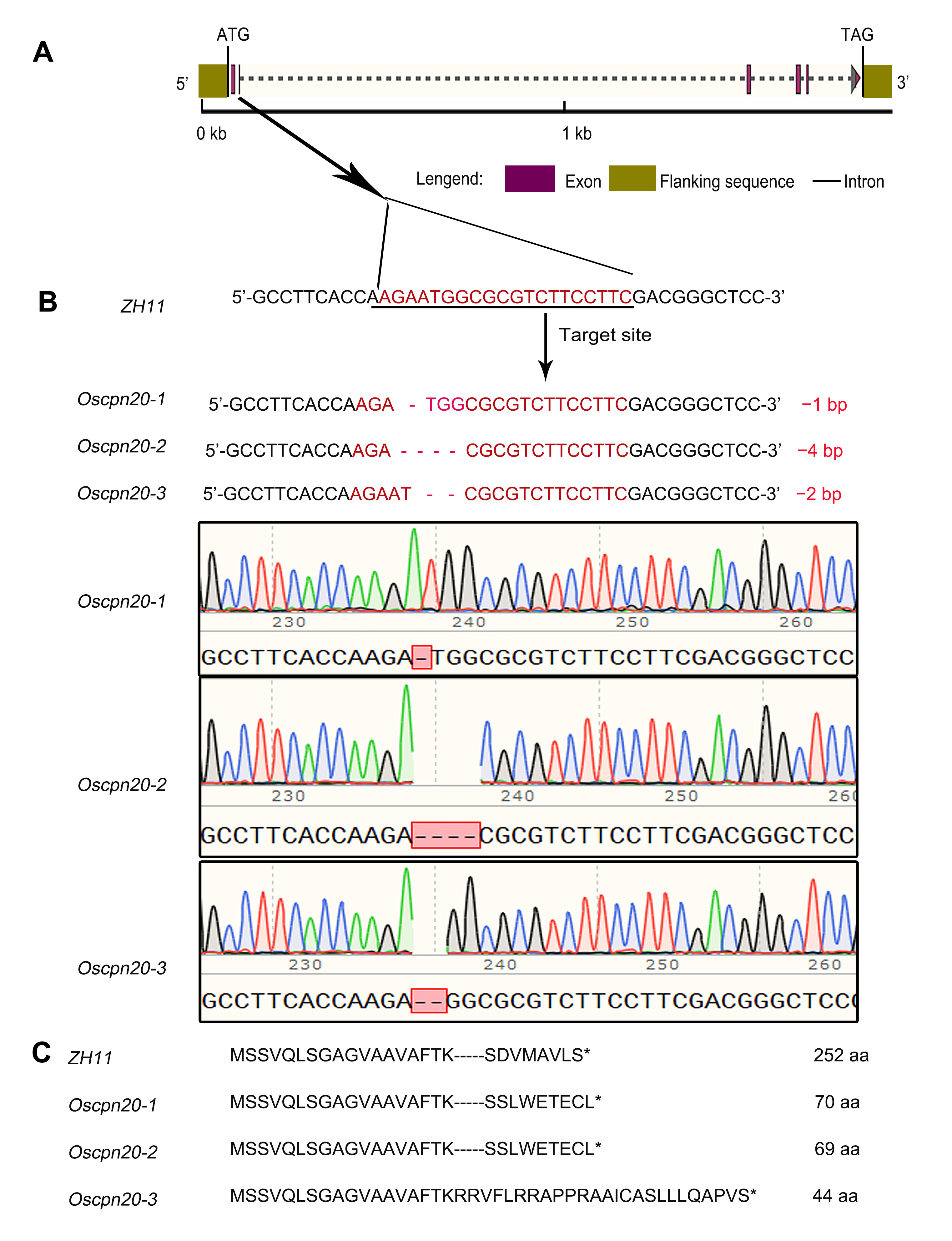


**Figure S12.** **Genotyping of *oscpn20* mutants in ZH11 background**

**(A)** Target sites of Clustered Regularly Interspaced Short Palindromic Repeats (CRISPR)/CRISPR-associated protein 9 (Cas9) were both designed on the first exon of *OsCPN20* in ZH11 background. **(B)** *OsCPN20* base sequence alignment between the wild type (ZH11) and *Oscpn20* mutants. Target sites are marked in red. Sequences in boxes are the target sequences in ZH11. Inserted nucleotides are indicated with red uppercase letters. **(C)** OsCPN20 protein sequence alignment between ZH11 and *Oscpn20* mutants.


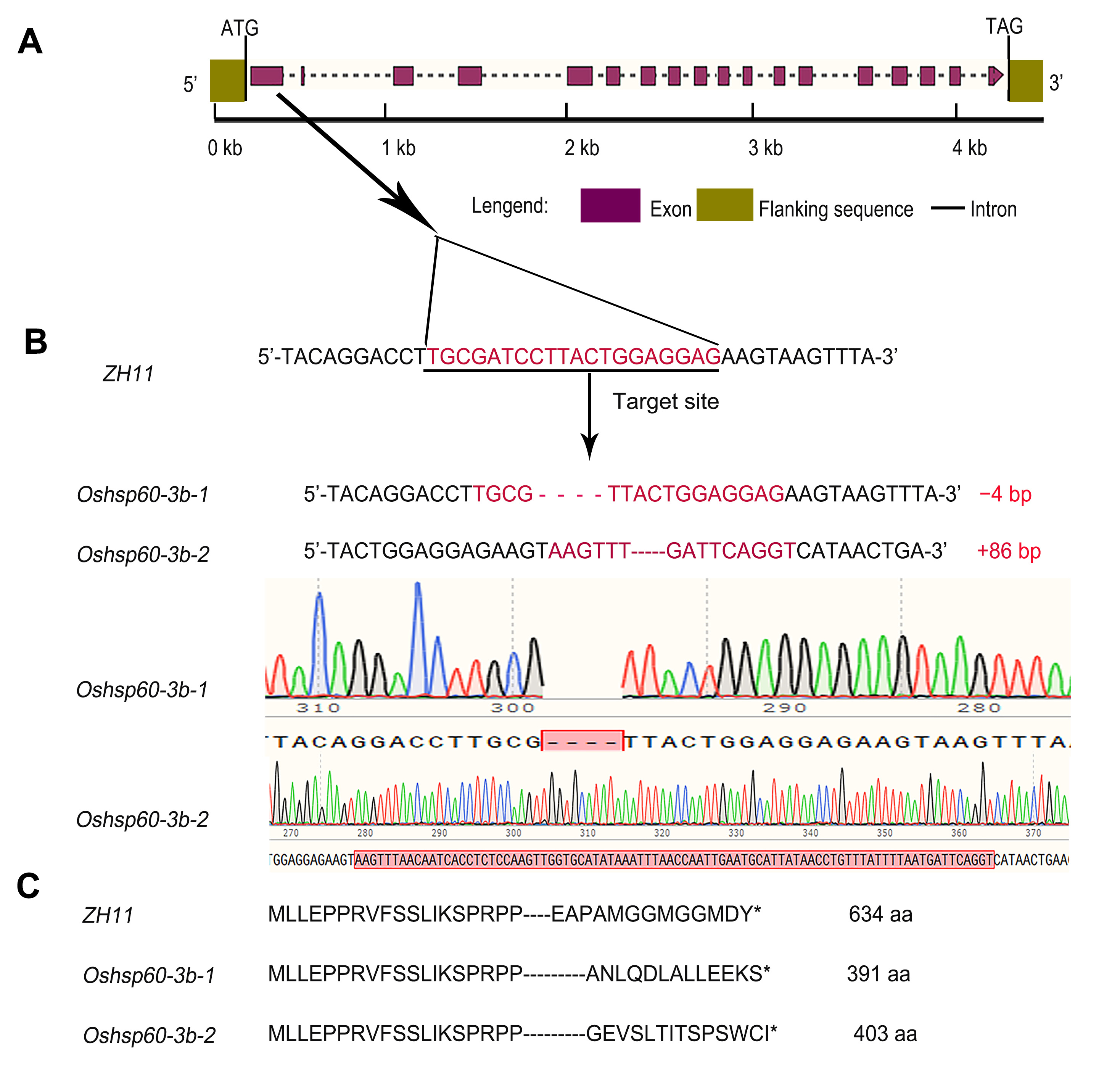


**Figure S13.** **Genotyping of *oshsp60-3b* mutants in ZH11 background**

**(A)** Target sites of CRISPR/Cas9 were both designed on the first exon of *OsHSP60-3B*. **(B)** *OsHSP60-3B* base sequence alignment between ZH11 and *Oshsp60-3b* knockout lines. Target sites are marked in red. Sequences in boxes are the target sequences in ZH11. Inserted nucleotides are indicated with red uppercase letters. **(C)** OsHSP60-3B protein sequence alignment between ZH11 and *Oshsp60-3b* mutants.

**Table S1.** **Sequences of all primers used in this study**

| Primers | Primer sequences (5′ → 3′) |
| --- | --- |
| OsCPN10a-qPCR-1F | AATTCTGCAAAAGTAGTGGCTG |
| OsCPN10a-qPCR-1R | TCAGTTCCTCCATACTCAGGTA |
| OsCPN10a-cs-F | gtcgatgaaccgggtgctgg |
| OsCPN10a-cs-R | cctgcttggtggtctccggg |
| OsCPN10a-seq-F | CCCGTCACCCACTCACCCGTCT |
| OsCPN10a-seq-R | CACCGCTCTATGTCCCAAACCTC |
| conum-sfu2af-F | AGGAGCCATGGGAGAAGC |
| conum-sfu2af-R | GTTCACGGCGGAAATCAT |
| conum-HYG-F | CGCATAACAGCGGTCATT |
| conum-HYG-R | CTGCTCCATACAAGCCAAC |
| proOsCPN10a-WGL-F | cccgggtgagctcggtaccttgaaacactcccagtgatattagg |
| proOsCPN10a-WGL-R | tcagatctaccatctagtaagcttcgacgggatcagcctcct |
| OsCPN10a-pro-ver-F | ggtttacctattctaccctcacctag |
| proOsCPN10a-1824-F | ttgaaacactcccagtgatattagg |
| proOsCPN10a-4136-R | cgacgggatcagcctcct |
| OsCPN10a -CDS-1F | TCACCCGTCTCCTCCACGCCCATC |
| OsCPN10a -CDS-1R | CTCCTCGAGTCGAGCCGTCCAAGT |
| OsCPN10a -CDS-2F | CGCCCATCAACTTGCTCCTCAAGA |
| OsCPN10a -WLG-Kpn-F | GGATCCCCGGGTGAGCTCGGTACCatggcggcgaggaggctga |
| OsCPN10a -WLG-Hind-R | AGCGGCCGCACTAGTAAGCTTctcctcaagccgtccgagt |
| OsCPN10a-C5x-BamHIII | CGCGATATCGTCGACGGATCCatggcggcgaggaggctga |
| OsCPN10a-C5x-HindIII | CTTTCGTTTTATTTGAAGCTTctcctcaagccgtccgagt |
| OsCPN10a-6p-BamHIII | TTCCAGGGGCCCCTGGGATCCatggcggcgaggaggctga |
| OsCPN10a-6p-XhoI | GTCACGATGCGGCCGCTCGAGctcctcaagccgtccgagt |
| OsCPN10b-CDS | atggcgaggcggctgatcccgt |
| OsCPN10b-CDS | tccacaagggtgcccaag |
| OsCPN20-CDS | atgtcgtcggtgcagctct |
| OsCPN20-CDS | agataggacagccatcacatc |
| OsCPN20-WLG-Kpn-F | GGATCCCCGGGTGAGCTCGGTACCatgtcgtcggtgcagctct |
| OsCPN20-WLG-Hind-R | AGCGGCCGCACTAGTAAGCTTagataggacagccatcacatc |
| OsCPN10b-WLG-Kpn-F | GGATCCCCGGGTGAGCTCGGTACCatggcgaggcggctgatcccgt |
| OsCPN10b-WLG-Hind-R | AGCGGCCGCACTAGTAAGCTTtccacaagggtgcccaag |
| OsHSP60-3B-CDS | atgcttctagagcctcctcgcg |
| OsHSP60-3B-CDS | gtaatccattccacccatgcct |
| OsCPN20-6p-BamHIII | TTCCAGGGGCCCCTGGGATCCatgtcgtcggtgcagctct |
| OsCPN20-6p-XhoI | GTCACGATGCGGCCGCTCGAGagataggacagccatcacatc |
| Oscpn10b-6p-BamHIII | TTCCAGGGGCCCCTGGGATCCatggcgaggcggctgatcccgt |
| Oscpn10b-6p-XhoI | GTCACGATGCGGCCGCTCGAGgtccacaagggtgcccaag |
| OsHSP60-6p-BamHI-F | TTCCAGGGGCCCCTGGGATCCatgcttctagagcctcctcg |
| OsHSP60-6p-XhoI-R | GTCACGATGCGGCCGCTCGAGgtaatccattccacccatgc |
| OsHSP60-head-R | gacccattgtcactttgactgc |
| OsHSP60-3B-WLG-Kpn-F | GGATCCCCGGGTGAGCTCGGTACCatgcttctagagcctcctcgcg |
| OsHSP60-3B-WLG-Hind-R | AGCGGCCGCACTAGTAAGCTTgtaatccattccacccatgcct |
| OsABIL1-WLG-Kpn-F | ggatccccgggtgagctcggtaccatggaggacgtggcggtggc |
| OsABIL1-WLG-Hind-R | agcggccgcactagtaagcttcttgcaagcaaaaattaatt |
| OsABIL1-C5x-BamHI-F | cgcgatatcgtcgacggatccATGGTGTTCGAGGATTCG |
| OsABIL1-C5x-HindIII-R | ctttcgttttatttgaagcttCGACAAGCACTCAGCAGCT |
| OsABIL1-AD-F | GCCATGGAGGCCAGTGAATTCATGGTGTTCGAGGATTCG |
| OsABIL1-AD-R | ATGCCCACCCGGGTGGAATTCCGACAAGCACTCAGCAGCT |
| OsPYL10-WLG-Kpn-F | GGATCCCCGGGTGAGCTCGGTACCatggagcagcaggaggaa |
| OsPYL10-WLG-Hind-R | AGCGGCCGCACTAGTAAGCTTttccgccgccgccggtgg |
| OsPYL10-C5x-BamHI-F | CGCGATATCGTCGACGGATCCatggagcagcaggaggaa |
| OsPYL10-C5x-HindIII-R | CTTTCGTTTTATTTGAAGCTTttccgccgccgccggtgg |
| OsPYL10-6p-BamHI-F | TTCCAGGGGCCCCTGGGATCCatggagcagcaggaggaa |
| OsPYL10-6p-XhoI-R | GTCACGATGCGGCCGCTCGActtttccgccgccgccggtgg |
| OsZEP15-qPCR-F | TCG​ACG​ACG​AGA​TGG​AGA​AG |
| OsZEP15-qPCR-R | GCG​GCG​GTA​GTT​GAT​GAT​GT |
| OsZEP182-qPCR-F | CGA​GGA​GAA​GGA​GGA​GGA​GAT |
| OsZEP182-qPCR-R | GCG​GCG​GTA​GTT​GAT​GAT​GT |
| OsNCED1-qPCR-F | CTCACCATGAAGTCCATGAGGCTT |
| OsNCED1-qPCR-R | GTTCTCGTAGTCTTGGTCTTGGCT |
| OsNCED4-qPCR-F | TCGGGAGGTACGACTTCCAT |
| OsNCED4-qPCR-R | TTGAGGTACGGCTTGGACAC |
| OsNCED3-qPCR-F | CCCTCCCAAACCATCCAAACCGA |
| OsNCED3-qPCR-R | TGTGAGCATATCCTGGCGTCGTGA |
| OsNCED5-qPCR-F | ACATCCGAGCTCCTCGTCGTGAA |
| OsNCED5-qPCR-R | TTGGAAGGTGTTTTGGAATGAACCA |
| OsABI5-qPCR-F | AGCGGTGAACCAGTTTGATT |
| OsABI5-qPCR-R | ATCTGCCTGTTTCCTCTCCA |
| OsABA8ox2-qPCR-F | GCGAGACGCTCCAGCTCTA |
| OsABA8ox2-qPCR-R | ACGCCTAAGCCTGCTGGTT |
| OsABA8ox1-qPCR-F | TCGATGGGGTGGCCGTACGTC |
| OsABA8ox1-qPCR-R | ACTTGTTCCGCTTCTTGTTGAA |
| OsABA8ox3-qPCR-F | ACGCCTAAGCCTGCTGGTT |
| OsABA8ox3-qPCR-R | TGCTGTTGCGGCGCAGTCG |
| OsPYL10-qPCR-F | CAAGGATGAGACATGCTACTTCGT |
| OsPYL10-qPCR-R | AGGCAGGCCAGACTTAACAT |
| OsPYL4-qPCR-F | TACTCCTCCATCCTGACCGT |
| OsPYL4-qPCR-R | GCGTTCAGAAACCTCTGCAAG |
| OsPP2C68-qPCR-F | GCT​GCT​GAT​GAG​GTT​GTT​GAG |
| OsPP2C68-qPCR-R | AGC​TGC​AAG​AAC​CGA​GAT​GA |
| OsPP2C30-qPCR-F | CGGCGAGTTCAAGAACAGCA |
| OsPP2C30-qPCR-R | CGTCGTCGTCGTAGTTCCTG |
| OsABF1-qPCR-F | CTGATGGATCCGATGGACCG |
| OsABF1-qPCR-R | GCAACTAGGCTACTGCCGAT |
| OsCPN10a-MT1-F | AATAATGGTCTCAGGCGTCGATGAACCGGGTGCTGGGTTTTAGAGCTAGAAATAGC |
| PCBC-MT1-R | ATTATTGGTCTCTGCTTCTTGGTGCCGC |
| OsCPN10a-MT2-F | ATATATGGTCTCaAAGCCCCGGAGACCACCAAGCAGGGTTTTAGAGCTAGAAATAGC |
| PCBC-MT2(m6b)-R | AATAATGGTCTCaAACACAAGCGGCAGCGCGCG |
| OsCPN20-MT3(m6b)-F | ATATATGGTCTCaTGTTGACTAGAGACGCTTGATTGTGTTTTAGAGCTAGAAATAGC |
| OsCPN20-MT4-R | ATTATTGGTCTCTAAACATCTGCAAGCTGACCTCAACGCCACGGATCATCTGCACAACT |
| OsHSP60-3B-MT3(m6b)-F | ATATATGGTCTCaTGTTGCCTCCAAGGCTCGGCAAGCGTTTTAGAGCTAGAAATAGC |
| OsHSP60-3B-MT4-R | ATTATTGGTCTCTAAACATAACCACATTGCGTCCCTTGCCACGGATCATCTGCACAACT |
| OsCpn10-AD-F | gccatggaggccagtgaattcATGGCGGCGAGGAGGCTG |
| OsCpn10-AD-R | atgcccacccgggtggaattcTTACTCCTCAAGCCGTCCGA |
| OsCPN20-BD-F | atggccatggaggccgaattcATGTCGTCGGTGCAGCTCTC |
| OsCPN20-BD-R | tcgacggatccccgggaattcTCAAGATAGGACAGCCATCACATC |
| OsCPN10b-BD-F | atggccatggaggccgaattcATGGCGAGGCGGCTGATC |
| OsCPN10b-BD-R | tcgacggatccccgggaattcTCAGTCCACAAGGGTGCCC |
| OsHSP60-BD-F | atggccatggaggccgaattcCATGTACCGCGCGGCCGCCA |
| OsHSP60-BD-R | tcgacggatccccgggaattcTTAGTAATCCATTCCACCCATGC |
| OsPYL10-BD-F | atggccatggaggccgaattcatggagcagcaggaggaa |
| OsPYL10-BD-R | tcgacggatccccgggaattcttccgccgccgccggtgg |
| OsCPN20-Seq-F | TGAGGAGTATTGTTGGCGTCTG |
| OsCPN20-Seq-R | CTTCACAAGCACTCTGTCTCCC |
| OsHSP60-Seq-F | TATTGTGGTGGTGCTACTGA |
| OsHSP60-Seq-R | AGCGGACAAAAGATACAC |
| OsCPN20-qPCR-F | CCCAAAGTATACCTCGCTCAAG |
| OsCPN20-qPCR-R | GAATCCCACCAACAGTCTTCTC |
| OsHSP60-3B-qPCR-F | CGAGGTGGAGAAGGTGAAGG |
| OsHSP60-3B-qPCR-R | TGATGAGGTTGATGCGGTTG |
| OsCPN10b-Seq-F | TCGCCTCCACCTTAAAACCC |
| OsCPN10b-Seq-R | AACCTGCTTGGACGTCTCAG |
| OsCPN20-Seq-F | AACCATAACCAAGCTCGCCA |
| OsCPN20-Seq-R | AATCGAGGATGTCGTCGGTG |
| OsHSP60-3B-Seq-F | CCCAACATCTGAGGCTCGAA |
| OsHSP60-3B-Seq-R | TGTTGTTCAGGTCTGTGCTGT |
